# Ancestral Protein Reconstruction Uncovers a Thermotolerant Rieske Oxygenase with Enhanced *O*‑Demethylation Activity toward 3‑*O*‑Methylgallate

**DOI:** 10.1021/acssynbio.6c00325

**Published:** 2026-06-03

**Authors:** Augusto Rodrigues Lima, Gabriel Gonçalves Dias, Samuel J. Davis, Gabriela de Lima Menezes, Adriano Freitas Lima, Mariany da Silva Costa, Lúcia Daniela Wolf, Fernanda Miyuki Kashiwagi, Mariana Abrahão Bueno Morais, Mikael Bodén, Gerhard Schenk, Priscila Oliveira de Giuseppe

**Affiliations:** † Brazilian Biorenewables National Laboratory (LNBR), 215006 Brazilian Center for Research in Energy and Materials (CNPEM), Campinas, São Paulo 13083-100, Brazil; ‡ Graduate Program in Genetics and Molecular Biology (PPG-GBM), Institute of Biology, State University of Campinas (UNICAMP), Campinas, São Paulo 13083-970, Brazil; § School of Chemistry and Molecular Biosciences, 1974 The University of Queensland, St Lucia, Queensland 4072, Australia

**Keywords:** ancestral sequence reconstruction (ASR), VanAB system, Rieske oxygenase, *O*-demethylation, lignin valorization, enzyme engineering, molecular dynamics simulations

## Abstract

Biological lignin valorization offers a renewable route to value-added chemicals, yet its industrial implementation is bottlenecked by the inherent instability and low efficiency of *O*-demethylating enzymes. Here, we employ ancestral sequence reconstruction (ASR) to resurrect highly stable and efficient variants of VanA-type Rieske nonheme iron oxygenases, which are notoriously difficult to engineer. Our lead ancestral variant, *Anc*VanA3, exhibits an unprecedented ∼30 °C increase in melting temperature (67 °C) and a 7-fold improvement in soluble yield compared to the extant *Xac*VanA. Crucially, *Anc*VanA3 achieves superior catalytic conversion of the recalcitrant lignin monomer 3-*O*-methylgallate (3OMG), reaching 92% conversion in the syringate-to-gallate funneling pathway. Molecular dynamics simulations reveal that this enhanced activity stems from the disruption of a gating mechanism involving a V300 gate-keeper residue, resulting in a constitutively open and enlarged substrate-binding pocket. Most significantly, biophysical characterization via mass photometry reveals that these enzymes exist predominantly as monomers in solution. This finding challenges the prevailing view that Rieske oxygenases self-assemble into intrinsically stable homotrimers to facilitate intersubunit electron transfer. Collectively, these results provide both a robust technological platform for lignin upgrading and a new mechanistic framework for the study of electron transfer in bioinorganic catalysis.

## Graphical abstract

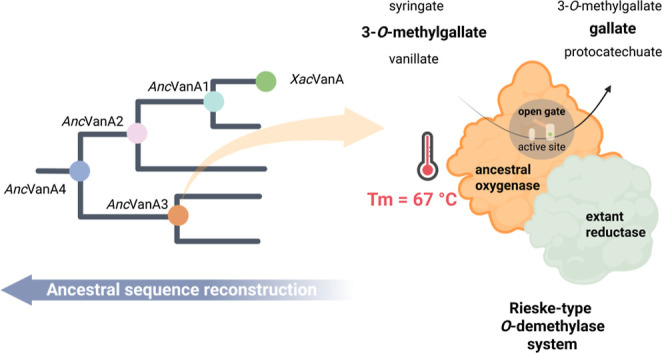

## Introduction

Lignin is the most abundant renewable source of aromatic carbon on Earth and a promising feedstock for the sustainable production of chemicals, fuels, and materials.^1–3^ However, its industrial potential remains largely underexploited due to its recalcitrance and structural heterogeneity, which hamper both its fragmentation and upgrading.^3–5^ Developing microbial and enzymatic systems capable of funneling diverse lignin-derived compounds into a single value-added product, thus simplifying downstream purification, is therefore a promising strategy toward integrating lignin-to-chemicals valorization into biorefineries.^1,4,5^

Several bacteria, including *Pseudomonas putida*, *Rhodococcus jostii* and *Amycolatopsis* spp. have emerged as promising chassis for converting lignin-derived aromatics into bioproducts such as muconic acid and polyhydroxyalkanoates.^6,7^ Within their metabolic funneling pathways, *O*-demethylation is a critical reaction and often represents a bottleneck, particularly in hosts such as *P. putida* KT2440 that are natively inefficient for this step.^7,8^

Three mechanistic classes of bacterial *O-*demethylases have been described to date: cytochrome P450 monooxygenases, tetrahydrofolate (THF)-dependent enzymes, and Rieske nonheme iron oxygenases.^9^ The first genetic descriptions of Rieske *O-*demethylases involved in the catabolism of lignin-derived aromatics emerged from studies of vanillate degradation by *Pseudomonas*, leading to the designation of VanA (the oxygenase component) and VanB (the reductase component).^10,11^ Despite their name, VanAB systems frequently act on methoxylated phenolics beyond vanillate, including syringate, 3-*O*-methylgallate (3OMG), and related benzoates.^12,13^ Additional Rieske *O-*demethylases include LigXacd^14,15^ from *Sphingobium* sp. SYK-6, involved in 5,5′-dehydrodivanillate *O*-demethylation, and the guaiacol-*O*-demethylases (GdmABs)^16^ from *Cupriavidus necator*, *Novosphingobium aromaticivorans* and *Sphingomonas wittichii*.

Although genome databases contain thousands of putative *vanAB* loci, experimental characterization remains limited to a few genera, including *Pseudomonas*,^10,11,17^ *Acinetobacter*,^18^ *Corynebacterium*,^19,20^ *Rhodococcus*,^21,22^ *Streptomyces*,^23^ and *Comamonas*.^24^ Recently, our group identified and characterized a VanAB system from the plant pathogen *Xanthomonas citri* (*Xac*VanAB), which demethylates vanillate, syringate, and 3OMG, expanding the known diversity of Rieske *O*-demethylases associated with lignin catabolism.^25^

Despite their relevance, the structural basis of substrate specificity and catalysis in Rieske *O*-demethylases remains poorly understood, and detailed in vitro steady-state kinetic parameters have been reported for only a limited number of systems, including VanAB from *P. putida* ^17^ and GdmAB^16^ systems. Although more than 20 crystal structures have been reported for Rieske oxygenases,^26^ only two are described as *O*-demethylases,^16,27^ with activity on phenolic compounds other than vanillate, syringate or 3OMG. No experimental structure has yet been determined for a VanAB system. In general, structural and mechanistic analysis of Rieske oxygenases is hindered by challenges including, lability of the mononuclear Fe site in these enzymes, incomplete cofactor incorporation, misfolding during heterologous expression, instability after purification, and O_2_ uncoupling.^12,26,28,29^ These limitations have prevented efforts to rationally engineer Rieske *O*-demethylases with improved stability or altered substrate selectivity.

Ancestral sequence reconstruction (ASR) provides a promising strategy to overcome these challenges.^30–32^ ASR is a computational technique that infers ancient protein sequences by applying evolutionary models to phylogenies of extant homologues.^31,33,34^ Resurrected ancestral enzymes frequently display enhanced properties, such as enhanced catalytic activity,^35–37^ increased thermostability,^37–40^ improved expression yield or solubility,^41,42^ and broader substrate promiscuity,^43–45^ traits that make them attractive for biotechnological applications.

In this study, we used ASR to generate and characterize four ancestral Rieske *O*-demethylases. One variant showed substantially increased thermal stability, formed a functional complex with the *Xac*VanB reductase, and displayed enhanced activity toward 3OMG. Computational analyses suggested that predicted ancient sequence alterations reshaped regions governing active-site access and substrate-binding pocket volume, offering a structural basis for the improved performance. We also revisited the oligomeric state of VanA oxygenases, providing insights relevant to electron transfer in VanAB systems. Together, these findings highlight ASR as a promising strategy for improving the stability and catalytic properties of Rieske *O*-demethylases for lignin valorization.

## Results

### Ancestral Rieske *O*-Demethylases Exhibit Enhanced Solubility

To identify Rieske *O*-demethylases with improved solubility and stability, we reconstructed a comprehensive phylogeny from more than a thousand of *Xac*VanA homologues, revealing taxonomically distinct clades dominated by *Actinobacteria* and *Proteobacteria*. Next, we selected four ancestral nodes at key taxonomic splits and inferred their sequences from a joint reconstruction using the ASR software GRASP^46^ for subsequent resurrection and characterization (Figure 1). *Anc*VanA1 represents the last common ancestor (LCA) of all *Xanthomonas* sequences, including extant *Xac*VanA, while *Anc*VanA2 traces further back to the LCA of the entire*Proteobacteria* clade. *Anc*VanA3 was derived from a well-supported node representing the LCA for the majority of the *Actinobacteria* sequences, and *Anc*VanA4 represents the most ancient node in the phylogeny, serving as the root ancestor for all other selected variants (Figure 1).

**Figure 1:**
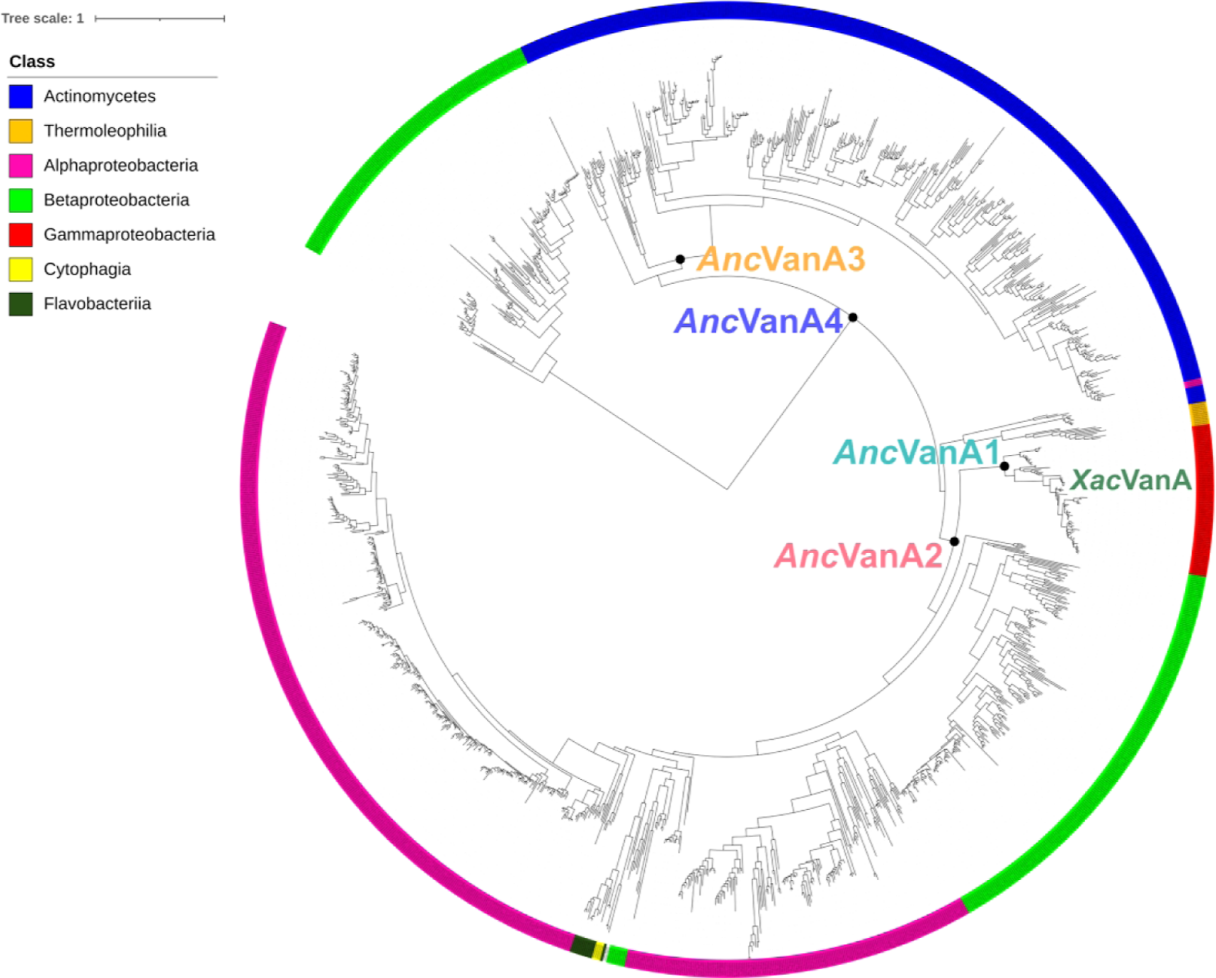
Phylogenetic tree of Rieske *O*-demethylases homologous to *Xac*VanA used for ancestral sequence reconstruction. Major taxonomic classes are indicated by the outer colored ring. The positions of the resurrected ancestral nodes (*Anc*VanA1–*Anc*VanA4) and the extant *Xac*VanA are highlighted. Tree scale bar indicates 1 substitution per site. The four ancestral variants selected for characterization represent strongly supported nodes, with ultrafast bootstrap approximations of at least 98%.

Heterologous expression in *Escherichia coli* followed by IMAC purification, buffer exchange and ∼15-fold protein concentration revealed substantially higher solubility for the ancestral enzymes compared to the extant *Xac*VanA. After this multistep procedure, the soluble yield of purified *Xac*VanA was 5 mg/L culture, whereas the ancestral variants yielded 11 mg/L (*Anc*VanA1), 20 mg/L (*Anc*VanA2), 34 mg/L (*Anc*VanA3), and 27 mg/L culture (*Anc*VanA4) (Figure S1). Consistent with these results, sequence-based solubility predictions using CamSol^47^ revealed a strong correlation between predicted solubility scores and experimentally measured soluble protein yields (Figure S2), supporting ancestral sequence reconstruction as a robust strategy for enhancing the solubility of Rieske *O*-demethylases.

### Resurrected Ancestral Enzymes Retain the *O*-Demethylase Function of *Xac*VanA but Exhibit Variable Performance Profiles

Since robust in vitro kinetic parameters could not be reliably determined for these enzymes, we evaluated in vivo performance, via whole-cell biocatalysis, in *E. coli* BL21 (DE3) expressing *Xac*VanA or its ancestral variants alongside the reductase *Xac*VanB. Activity required coexpression with *Xac*VanB, confirming that all ancestors retained the ability to form functional complexes with the extant reductase (Figures 2 and S3a). Conversely, a paralogous reductase from a VanAB-like system functionally divergent,^25^ yielded only residual activity, highlighting the critical role of coevolution in governing VanAB subunit compatibility (Figure S3b).

**Figure 2:**
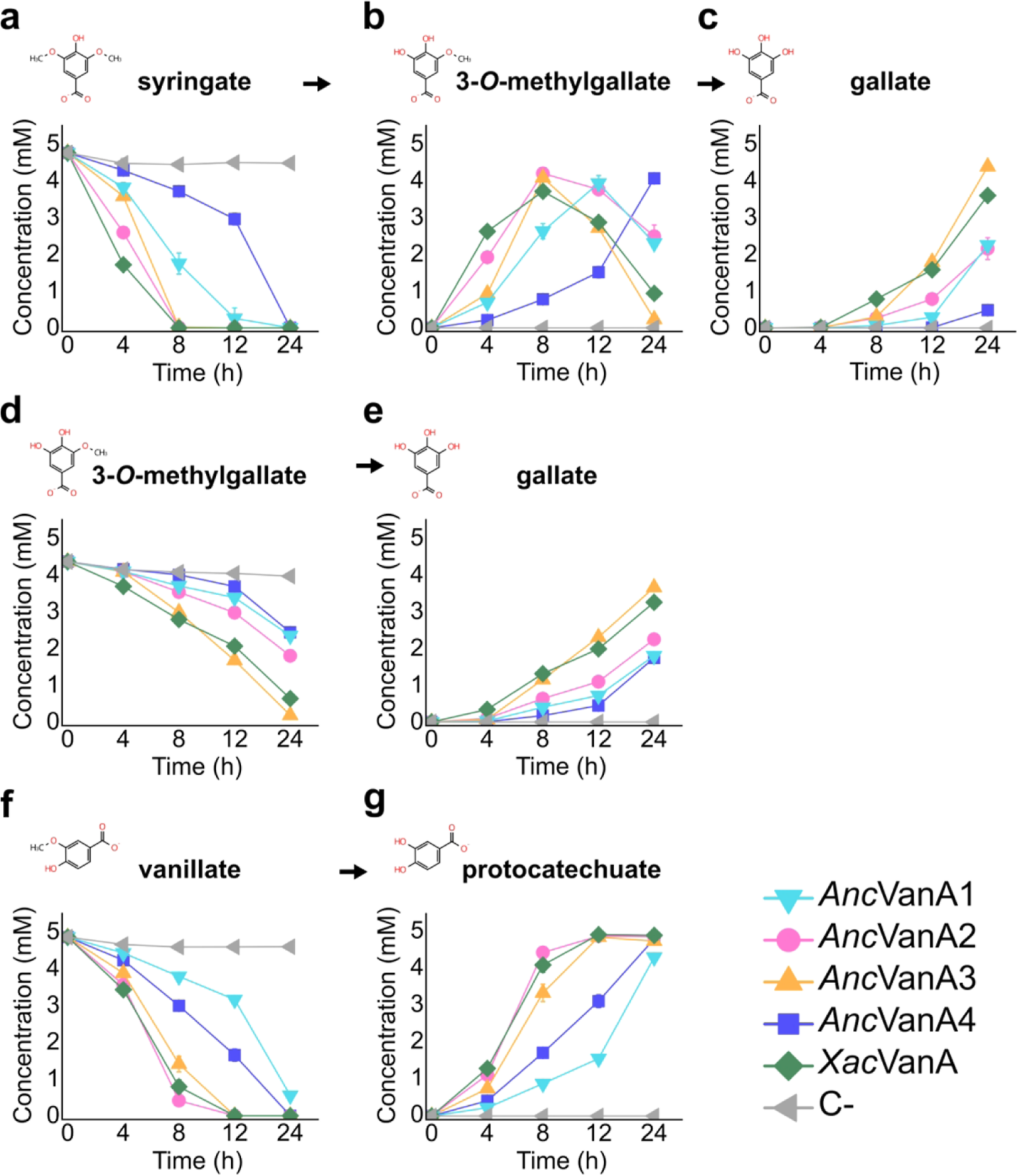
Whole-cell activity assays in *E. coli* show distinct substrate-dependent performance profiles for ancestral VanA variants. *E. coli* strains coexpressing VanA variants with the *Xac*VanB reductase were cultivated in M9 minimal medium supplemented with syringate, 3OMG or vanillate, as indicated. Culture supernatants were analyzed by HPLC to determine the concentrations of the aromatic compounds over time. (a) Syringate supplied as substrate. (b) 3OMG produced after the first *O*-demethylation of syringate. (c) Gallate produced after complete *O*-demethylation of syringate. (d) 3OMG supplied as substrate. (e) Gallate produced by *O*-demethylation of 3OMG. (f) Vanillate supplied as substrate. (g) Protocatechuate produced by *O*-demethylation of vanillate. Negative control (C−): strain expressing only the reductase *Xac*VanB. Data are presented as the mean ± SD of triplicates. Source data is available in Table S1.

With syringate as the substrate, the variants displayed distinct performance profiles. *Anc*VanA4 exhibited the slowest conversion, leaving substantial substrate in the first 12 h and accumulating the intermediate 3-*O*-methylgallate (3OMG) with minimal gallate formation, indicative of reduced efficiency in the two-step demethylation process (Figure 2a–c). *Anc*VanA1 showed the next lowest rate but achieved gallate levels comparable to *Anc*VanA2 within 24 h (∼45–47%) (Figure 2a–c and Table S1). Notably, while *Xac*VanA consumed syringate faster initially, *Anc*VanA3 surpassed the extant enzyme in the second demethylation step, achieving near-complete 3OMG depletion (Figure 2a,b). This resulted in a superior overall syringate-to-gallate conversion of 92%, compared to 76% for *Xac*VanA, indicating that *Anc*VanA3’s proficiency in the second step compensates for its slower initial rate (Figure 2a–c and Table S1).

When 3OMG was provided as the primary substrate, *Anc*VanA1 and *Anc*VanA4 remained the least efficient (∼40% gallate in 24 h), while *Anc*VanA2 showed moderate performance (Figure 2d,e). Consistent with the syringate data, *Anc*VanA3 achieved the highest efficiency, converting 84% of 3OMG to gallate, outperforming the extant *Xac*VanA (75%) and confirming its enhanced catalytic profile toward this intermediate (Figure 2d,e and Table S1). To evaluate the robustness and reproducibility of the superior performance of *Anc*VanA3 on 3OMG, we assessed its activity at different initial cell concentrations (used as a proxy for enzyme concentration). Under these tested conditions, *Anc*VanA3 outperformed *Xac*VanA in 3OMG demethylation, producing up to 5-fold more gallate than the extant enzyme (Supporting Information Figure S4). Together, these results indicate that the superior performance of *Anc*VanA3 is reproducible and robust across experimental conditions, reflecting a context-dependent but functionally relevant enhancement compared to the extant enzyme.

On vanillate, *Anc*VanA1 and *Anc*VanA4 again lagged behind, though *Anc*VanA4 reached full depletion by 24 h (Figure 2f,g). In contrast, *Anc*VanA2, *Anc*VanA3, and *Xac*VanA achieved complete consumption within 12 h. *Anc*VanA2 exhibited the fastest initial conversion, reaching 91% within 8 h, compared to 69% for *Anc*VanA3 (Figure 2f,g and Table S1).

Collectively, these results demonstrate that all four resurrected ancestors form functional complexes with *Xac*VanB and retain the *O*-demethylase activity of *Xac*VanA, while displaying distinct substrate-dependent performance profiles. Among the ancestral variants tested, *Anc*VanA2 exhibited the highest efficiency toward vanillate, whereas *Anc*VanA3 showed superior activity in the *O*-demethylation of 3OMG and in the two-step conversion of syringate to gallate.

### Ancestral Rieske *O*-Demethylases Exhibit Enhanced Thermal Stability with Variant-Specific Oligomeric Behavior

Given the enhanced catalytic performance of *Anc*VanA2 and *Anc*VanA3 in whole-cell assays, compared to the other ancestral variants, we next evaluated their thermal stability and assembly properties relative to the extant Rieske *O*-demethylase *Xac*VanA. Both ancestral enzymes exhibited a marked increase in thermal stability, with apparent average melting temperatures near 28 °C higher than that of the extant enzyme (Figures 3a and S5).

**Figure 3:**
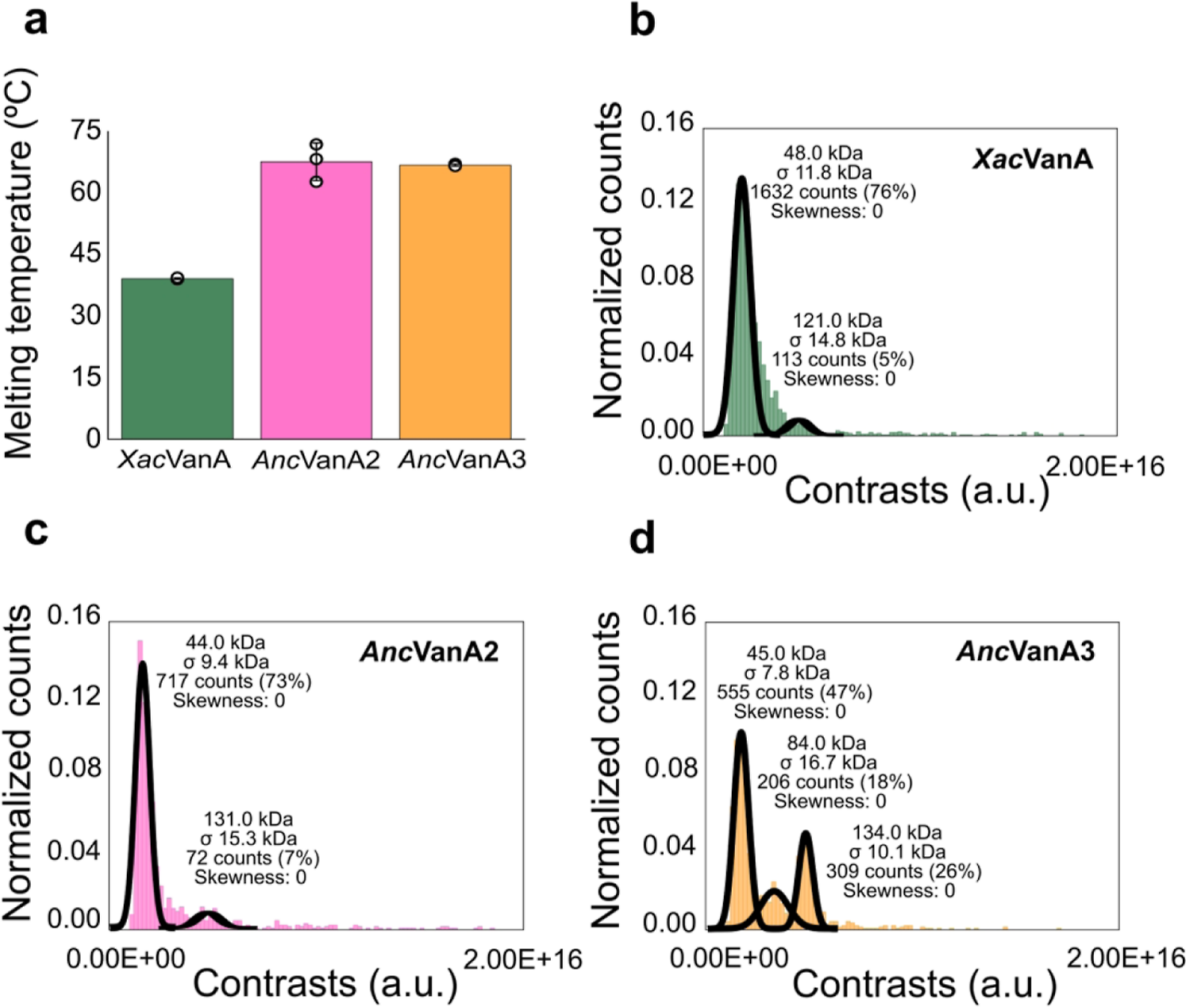
ASR yielded two thermotolerant VanA ancestors, one retaining the predominantly monomeric behavior of extant *Xac*VanA and the other exhibiting a shift in equilibrium toward trimer formation. Differential scanning fluorimetry (DSF) assays revealed that the two selected ancestral variants are more thermostable than the extant oxygenase *Xac*VanA (a). Mass photometry measurements showed similar particle population distributions for *Xac*VanA (b) and *Anc*VanA2 (c), whereas a distinct distribution pattern was observed for *Anc*VanA3 (d). Histograms represent the interferometric contrast obtained from mass photometry measurements, with the *x*-axis corresponding to particle contrast (arbitrary units), the primary signal measured in mass photometry, and the *y*-axis showing normalized event counts. The most intense peaks are consistent with the monomeric form of each protein, as inferred from sequence-based molecular weight predictions, while secondary peaks are consistent with dimeric and trimeric subpopulations, respectively.

To further investigate the structural basis underpinning these stability differences, the oligomeric states of *Xac*VanA, *Anc*VanA2 and *Anc*VanA3 were analyzed by mass photometry under near-physiological conditions (Figure 3b–d). Both *Xac*VanA and *Anc*VanA2 were predominantly monomeric in solution, accounting for 76% and 73% of the detected populations, respectively, with only minor trimeric subpopulations (5% and 7%). In contrast, *Anc*VanA3, while still mainly monomeric, exhibited a substantially increased trimeric fraction (26%). Consistent with these experimental findings, PDBePISA^48^ analysis of AlphaFold3 ^49^ predicted structures, which resemble the GdmA crystallographic trimer (Figure S6), indicated that the *Anc*VanA3 assembly may exhibit a larger buried interface area and a more favorable solvation free energy gain compared to *Xac*VanA and *Anc*VanA2, providing a structural rationale for the enhanced trimer formation observed experimentally (Figures 3b–d and S7).

Notably, *Anc*VanA3 also exhibited superior solubility and stability during purification and storage. Unlike *Xac*VanA and *Anc*VanA2, which rapidly precipitated during buffer exchange, concentration, or extended storage, *Anc*VanA3 remained soluble and could be reproducibly analyzed by SEC and SEC-MALS. These analyses revealed mixed oligomeric populations with a clear predominance of monomers, in agreement with mass photometry data (Figure S8).

Taken together, these results indicate that ASR not only improves thermal tolerance but can also reconfigure oligomerization equilibria in Rieske *O*-demethylases, thereby enhancing protein solubility and overall structural stability.

### *Xac*VanA and Ancestral Enzymes Conserve Canonical Rieske Motifs while Exhibiting a Noncanonical Substitution in a Substrate-Coordinating Residue

To gain further insight into the structural features underlying the distinct performance profiles of *Xac*VanA and its ancestral variants, we performed comparative sequence and structural analyses. These comparisons revealed strict conservation of the canonical 2Cys–2His motif in *Xac*VanA and its ancestral variants, a motif that is essential for coordination of the [2Fe–2S] Rieske cluster (Figures 4a and S9). Likewise, the residues involved in nonheme iron binding are fully conserved and follow the classical 2His–1Asp (carboxylate) coordination pattern^50^ (Figures 4a and S9).

**Figure 4:**
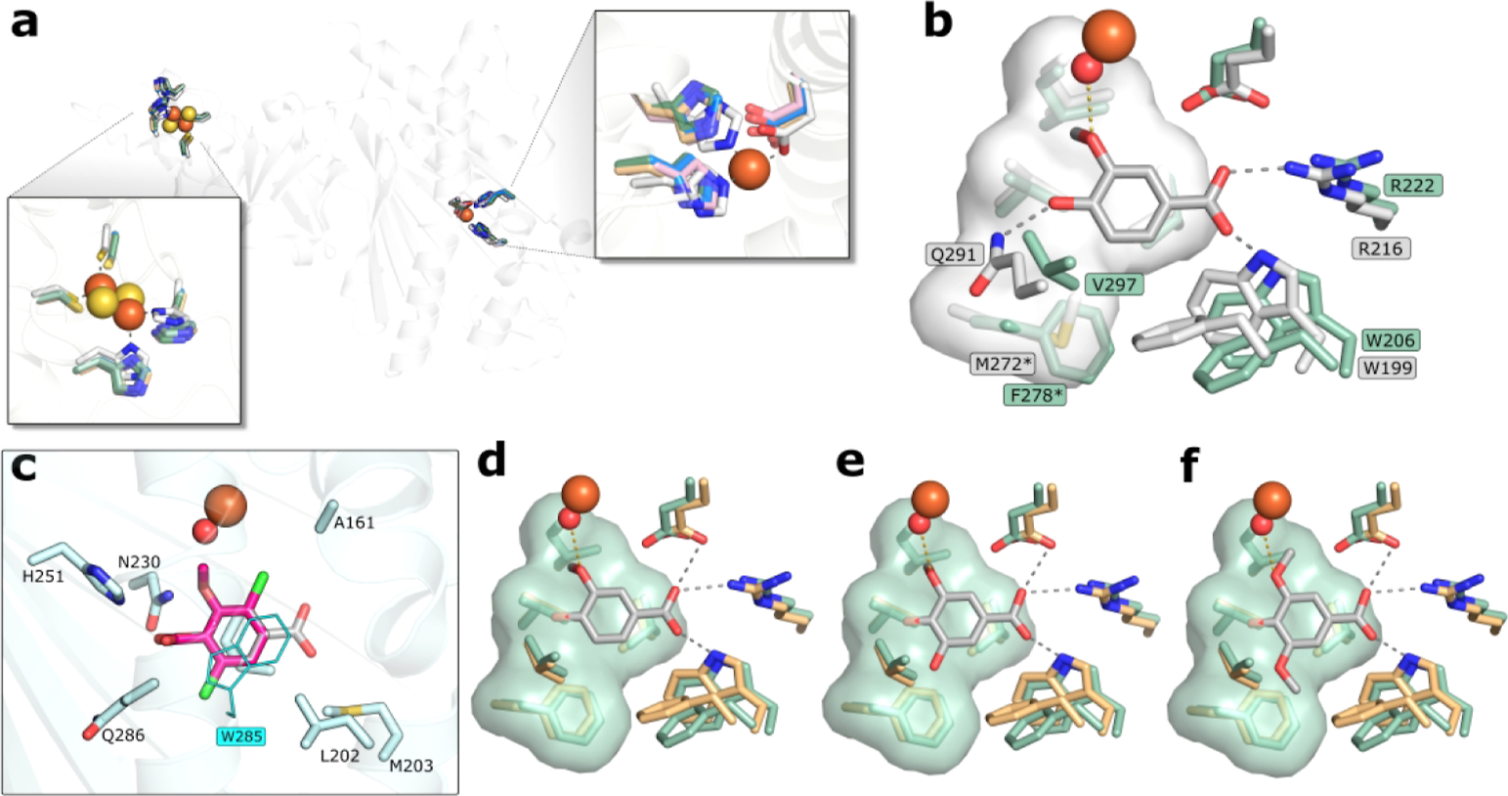
*Xac*VanA and *Anc*VanA3 conserve canonical Rieske motifs and share the same predicted substrate-binding residues. (a) Cartoon representation of DMO (white, PDB ID 3GL2) highlighting the conserved residues typical of Rieske oxygenases, showing the preservation of key positions in all proteins under investigation. *Anc*VanA1 (cyan C atoms), *Anc*VanA2 (pink C atoms), *Anc*VanA3 (light orange C atoms), *Anc*VanA4 (violet C atoms; occluded due to superposition), and *Xac*VanA (green C atoms). (b) Substrate-binding pocket comparison of *Ph*VanA (white C atoms) and *Xac*VanA (green C atoms) predicted models showing the conservation of most residues but the substitution Q291 V and M272F. Gray surface represents the hydrophobic surface of *Ph*VanA residues. (c) Active-site view of DMO (PDB ID 3GL2) showing the substrate binding position within DMO and the proposed location of molecular oxygen for catalysis, here represented by a crystallographic water (red sphere) close to the Fe^III^ (orange sphere). (d–f) Superposition of predicted models of *Anc*VanA3 (light orange C atoms) and *Xac*VanA (green C atoms) with DMO crystallographic model (PDB ID 3GL2) used as a guide for manual docking of vanillate, 3OMG and syringate, respectively. For clarity purposes, the DMO structure was omitted. Nitrogen and oxygen atoms are shown in blue and red, respectively. Dashed lines represent interatomic distances compatible with putative hydrogen bonds.

Two of the three residues previously suggested as forming hydrogen bonds with vanillate in VanA from *P. putida* HR199,^16^ namely W199 and R216, are also conserved in *Xac*VanA and the ancestral sequences (Figures 4b and S9). In contrast, the third predicted hydrogen-bonding residue (Q291) is replaced by a valine (Figures 4b and S9). The same Q291V substitution is observed in VanA sequences from *Corynebacterium glutamicum* ATCC 13032, *Corynebacterium efficiens* YS314 and *Rhodococcus ruber* R1 (Figure S9), indicating that *Xac*VanA and its ancestral variants share a feature characteristic of some Corynebacteriales Rieske *O*-demethylases.

To explore productive substrate orientations, we performed pair fitting of the six aromatic carbons of syringate, 3OMG and vanillate onto the aromatic ring of dicamba in the crystallographic structure of dicamba *O*-demethylase (DMO), orienting the methoxy-substituted carbon such that its methyl group points toward the crystallographic water molecule, likely representing the binding site of the molecular oxygen that is activated by the catalytic Fe^II^ during catalysis^27^ (Figure 4b–f). Interestingly, this analysis indicated that the putative substrate-binding sites of *Xac*VanA and *Anc*VanA3 are identical in amino acid composition and very similar in side chain conformation (Figure 4d–f). Despite the limitations of the docking strategy employed, all binding sites appeared compatible with the accommodation of vanillate, syringate, and 3OMG (Figure 4d–f). The same structural conservation of the putative substrate-binding residues was observed for the other ancestral variants (Figure S10), indicating that variations in other regions might explain the differences in catalytic performance observed between them in *E. coli* whole-cell assays.

The conserved substrate-binding site architecture shared between *Xac*VanA and *Anc*VanA3 led us to hypothesize that the superior performance of *Anc*VanA3 toward 3OMG demethylation may arise from differences in the dynamic behavior of the substrate-binding pocket. To test this hypothesis, we performed molecular dynamics (MD) simulations using AlphaFold2-predicted models for both proteins.

The MD trajectories revealed a pocket opening/closing behavior in the substrate-binding region of *Xac*VanA, which was not observed in *Anc*VanA3 (Figures 5a–e, Supporting Information 1). Two of three *Xac*VanA independent simulations sampled both conformational states, while the pocket in simulation 3 did not fully close during the 500 ns simulation (Figure S11). In contrast, all *Anc*VanA3 independent simulations consistently maintained an open conformation of the substrate-binding pocket throughout the simulation (Figure S12), which could facilitate substrate entry and product release.

**Figure 5:**
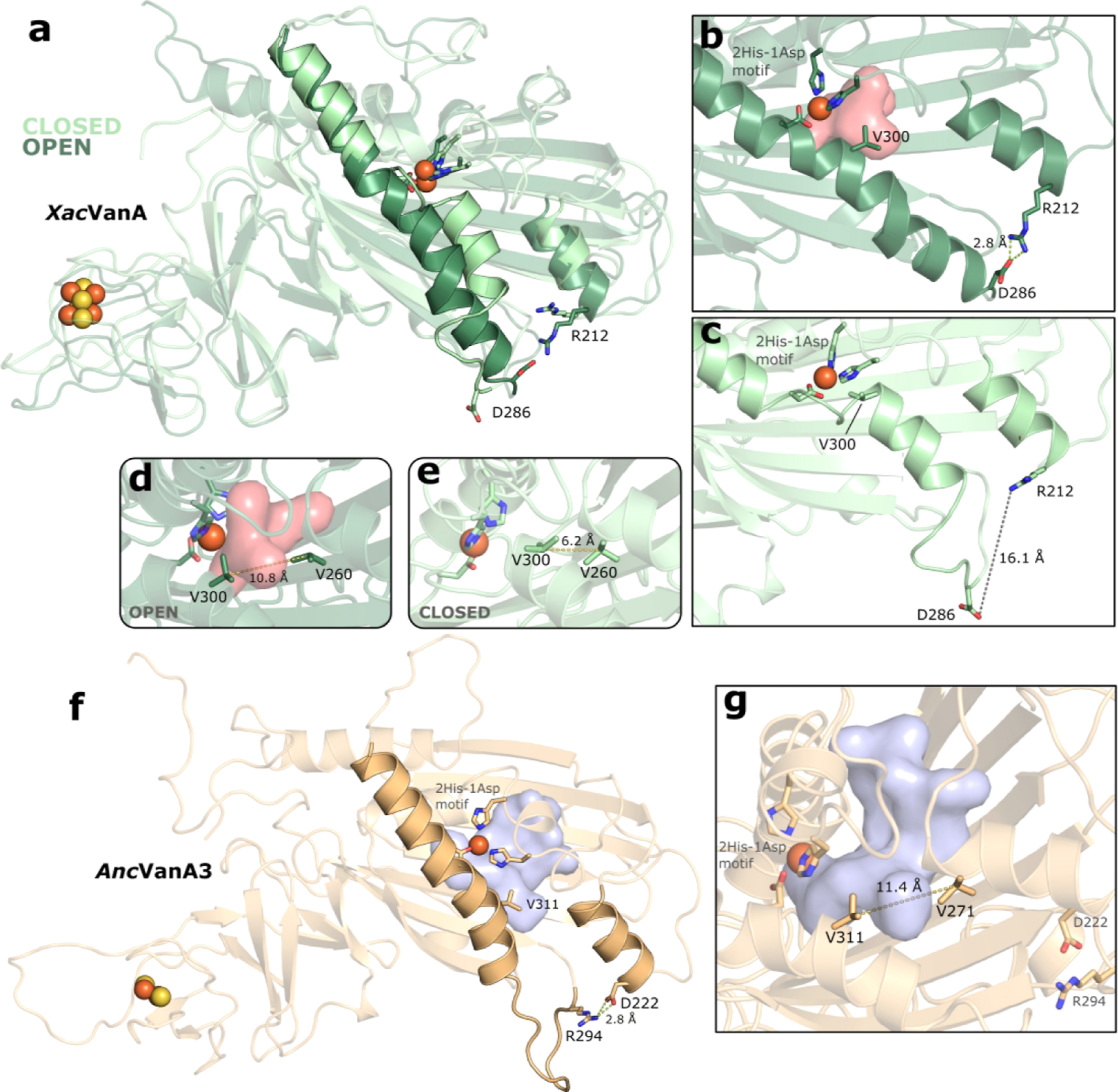
Differences in substrate-binding pocket dynamics in *Xac*VanA versus *Anc*VanA3. (a) Representative frames from MD simulations of *Xac*VanA illustrating open and closed substrate-binding pocket conformations. (b) Open conformation of *Xac*VanA emphasizing a predicted cavity (red surface) found when the salt bridge (R212-D286) is stabilized. (c) Closed conformation of *Xac*VanA highlighting the salt bridge (R212-D286) disruption, the V300 alternative conformation and structural rearrangement in a local helix. (d,e) Representative distances between V300 and V260 in *Xac*VanA for the open (d) and closed (e) conformations. (f) Representative MD simulation frame of *Anc*VanA3 highlighting a large cavity (blue surface) comprising the substrate-binding pocket. (g) Representative distance between V311 and V271 in *Anc*VanA3, correlating with the predicted enlarged cavity.

Analysis of global protein motions indicates that the transition between open and closed states correlates with both a conformational change of residue V300 in *Xac*VanA, located near the substrate-binding pocket, and the disruption of a distal salt bridge (Figures 5b–e and S11). The proximity of V300 to V260, used as a reference point for a deeper region of the pocket, tracks the helix movement associated with pocket occlusion. The observed V300 flip aligns with the analogous role of the unrelated W285 residue in the Rieske oxygenase DMO, where flipping of DMO_W285 is critical for substrate (dicamba) access.^27^ This analogy suggests that flipping of *Xac*VanA_V300 may similarly regulate active-site accessibility (Figure 5d,e).

In addition, the distance between the R212 and D286 side chains, located far from the substrate-binding site, correlates inversely with the substrate-pocket volume (Figure S11). When the salt bridge remains intact, the pocket stays open with a volume between 200 and 300 Å^3^ (Figure S11). However, disruption of this interaction coincides with the conformational change that leads to pocket occlusion, indicating that the R212–D286 pair may participate in the coordinated motions governing the open-to-closed transition of the substrate-binding site.

*Anc*VanA3 exhibited a substantially larger substrate-binding pocket volume across all MD independent simulations when compared to *Xac*VanA (Figures S11 and S12). The residues V311 and V271 (corresponding to *Xac*VanA V300 and V260, respectively) remained mostly distant from each other throughout the simulations, with no evidence of an *Anc*VanA3 V311 flip (Figure S12), thereby preserving the cavity volume consistently large in *Anc*VanA3 (Figures 5f,g and S12). Similar to *Xac*VanA, *Anc*VanA3 contains a distal arginine–aspartate residue pair (R294-D222); however, this pair forms persistent contacts throughout the MD trajectories and are not represented by the same corresponding positions in *Xac*VanA (Figures 5f and S12). Curiously, the long helix harboring V300 in *Xac*VanA is shorter in *Anc*VanA3 due to a local disorder at its *N*-terminus, which might contribute to impair the closing mechanism of the substrate-binding pocket, favoring the open conformation (Figure 5a,f). Together, these results indicate that amino acid variations in regions distal to the active site promotes a wider and more accessible substrate binding site in *Anc*VanA3, likely facilitating the demethylation of 3OMG.

In summary, these data indicate that despite sequence divergence, including the noncanonical Q291V substitution, the catalytic core and substrate-binding site residues remain strongly conserved across ancestral and extant VanA enzymes, consistent with retention of *O*-demethylase function and compatibility with vanillate, syringate, and 3OMG substrates. This conservation explains the preserved in vivo activity of ancestral enzymes and indicates that variant-specific performance differences may arise from multiple factors, including altered dynamics of residues shaping the volume and accessibility of the substrate-binding site, as supported by molecular dynamics.

### Ancestral and Extant VanA Variants Exhibit Divergent Behavior in *P. putida* KT2440

As *Anc*VanA3 emerged as the most promising ancestral variant, we next evaluated its performance in *P. putida* KT2440, one of the most well-established chassis for the bioconversion of lignin-derived compounds.^51^ To this end, *Anc*VanA3 and *Xac*VanB genes were cloned into a plasmid and constitutively expressed in *P. putida* KT2440. For comparison, we also included a strain coexpressing the native *P. putida* VanAB (*Pp*VanAB) and the extant *Xac*VanAB system.

The resulting KT2440 strains were cultivated in minimal medium supplemented with either syringate or 3OMG. Unexpectedly, the strain coexpressing *Anc*VanA3 and *Xac*VanB failed to efficiently demethylate either substrate, displaying a performance comparable to the negative control harboring only the chromosomal *vanAB* loci (Figure 6a,b). In contrast, the strain expressing *Xac*VanAB completely consumed all aromatic compounds within 24 h, similar to *Pp*VanAB, although it exhibited lower conversion rates during the first 12 h of cultivation (Figure 6a,b).

**Figure 6:**
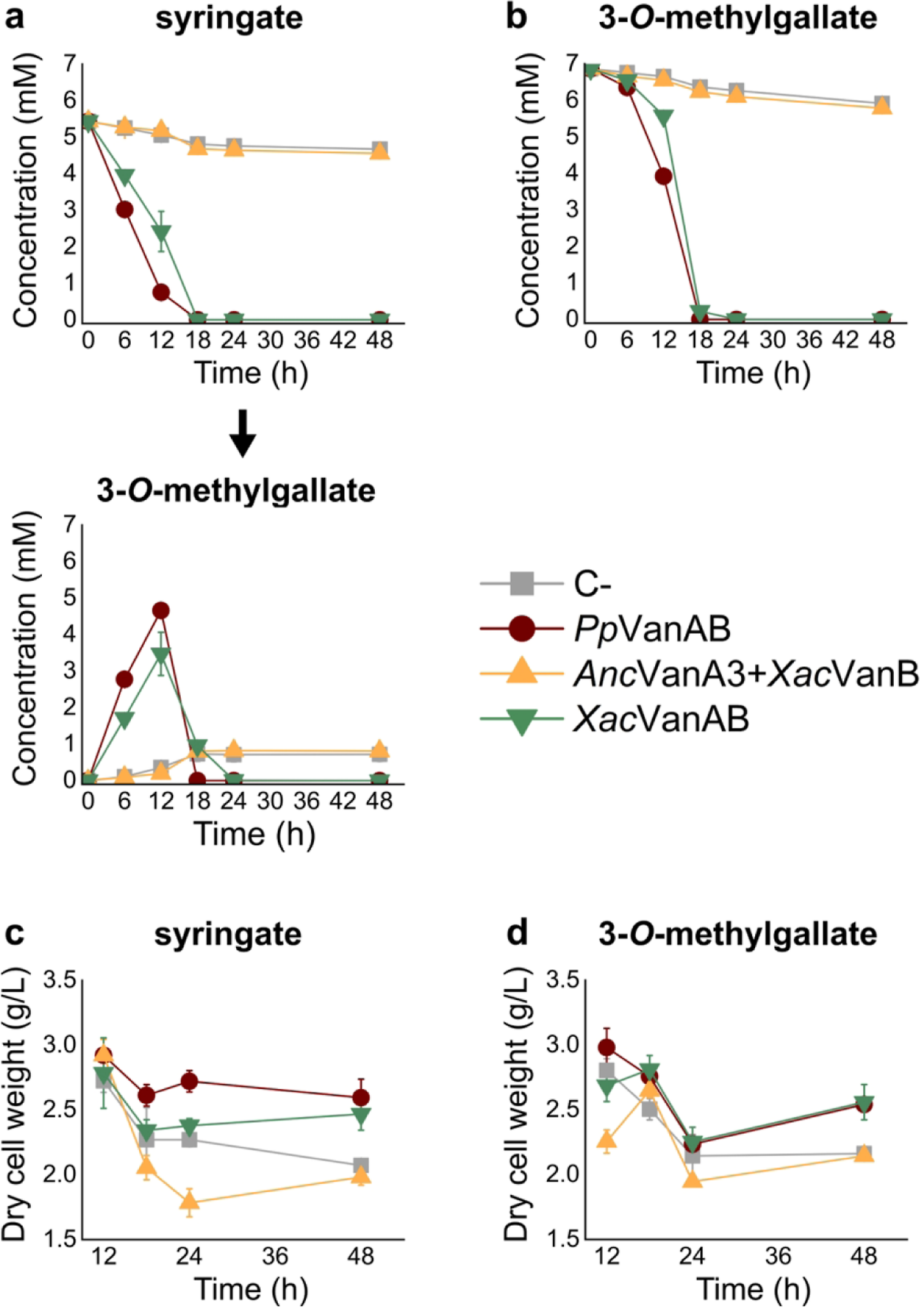
*Anc*VanA3 displays host incompatibility with *Pseudomonas putida* KT2440. *P. putida* KT2440 strains expressing the indicated plasmid-borne oxygenase–reductase combinations were cultivated in M9 minimal medium supplemented with aromatic substrates, as indicated. Culture supernatants were analyzed by HPLC to determine the concentrations of aromatic compounds over time. (a) Syringate supplied as substrate and 3OMG detected as the product of the first *O*-demethylation step. (b) 3OMG supplied as substrate. Gallate was not detected, consistent with its rapid consumption by *P. putida*. (c,d) Dry cell weight profiles monitored over time for the corresponding cultures; biomass measurements were initiated after 12 h of cultivation. Data are presented as the mean ± SD of triplicates. C–: negative control corresponding to *P. putida* KT2440 transformed with the empty plasmid. *Pp*VanAB: *P. putida* KT2440 expressing its native *vanA* and *vanB* genes from a plasmid-borne construct. *Anc*VanA3 + *Xac*VanB: *P. putida* KT2440 expressing the ancestral oxygenase *Anc*VanA3 together with the extant reductase *Xac*VanB from a plasmid-borne construct. *Xac*VanAB: *P. putida* KT2440 expressing the extant oxygenase *Xac*VanA and its cognate reductase *Xac*VanB from a plasmid-borne construct.

Because reliable optical density measurements could not be obtained due to distinct coloration profiles that developed across conditions, dry cell weight was used as a proxy for bacterial growth. Under syringate supplementation, the *Anc*VanA3-expressing culture exhibited a more pronounced reduction in dry cell weight between 12 and 24 h of cultivation compared with the other strains, whereas under 3OMG supplementation reduced dry cell weight was observed for this strain in most analyzed time points (Figure 6c,d). All strains depleted the glucose present in the medium within 12 h (Figure S13), indicating they were all metabolically active. These observations are consistent with *Anc*VanA3 expression imposing a cellular burden or toxicity in KT2440, potentially leading to reduced growth and plasmid instability, ultimately resulting in a loss of demethylation activity.

## Discussion

The extensive sequence diversity currently available in public databases necessitates computational strategies such as ASR to effectively explore the sequence space for high-performance enzymes. By targeting representative ancestral nodes, we successfully reduced a data set of over 1200 VanA-like sequences to four resurrected variants, prioritizing shared evolutionary lineages over broad sampling across the 141 identified genera. Notably, all reconstructed ancestors formed functional complexes with the extant *Xac*VanB reductase, indicating a high degree of conservation in the structural features required for VanAB interaction (Figure 7). Conversely, the lack of activity observed with a paralogous reductase from a functionally divergent system suggests that coevolutionary constraints at this interface govern subunit compatibility, implying that the efficiency of VanABs is critically dependent on the specificity of their interaction.

**Figure 7:**
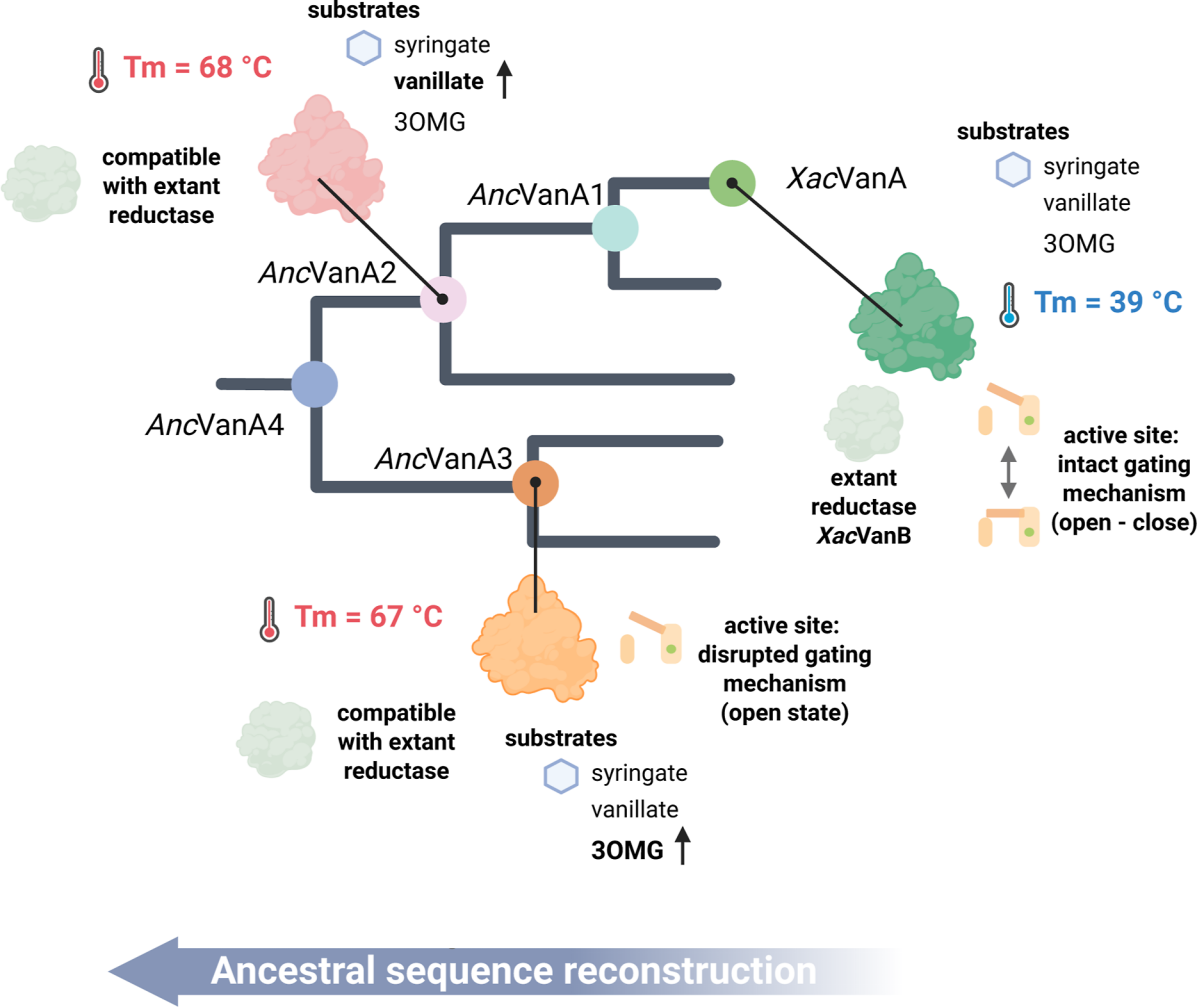
ASR applied to the VanA family revealed ancestral enzymes with superior thermotolerance and improved activity in a substrate-dependent manner. All ancestral variants formed active complexes with the extant reductase *Xac*VanB. The scheme highlights the variants displaying higher thermotolerance as well as superior activity toward vanillate (*Anc*VanA2) and 3OMG (*Anc*VanA3), respectively. Notably, the gating mechanism is disrupted in the *Anc*VanA3 variant, providing enhanced accessibility to the substrate-binding site and likely contributing to its higher efficiency in 3OMG conversion and in the two-step syringate-to-gallate pathway.

Beyond subunit compatibility, the resurrected variants displayed distinct, substrate-dependent performance profiles, which confirms that ASR successfully modulated their catalytic preferences (Figure 7). The *Actinobacterial* ancestor, *Anc*VanA3, exhibited a remarkable ∼28 °C increase in melting temperature and superior activity toward 3OMG, while the *Proteobacterial* ancestor, *Anc*VanA2, excelled in vanillate demethylation. Although the enhanced solubility of these ancestors may contribute to performance and confirms their superior structural stability, a hallmark of ASR,^31,32^ this feature alone does not explain the differential behavior observed across substrates. Instead, these substrate-dependent improvements likely stem from intrinsic alterations in substrate binding, transition-state stabilization, or product release.

A particularly unexpected finding from our biophysical characterization is that both the extant *Xac*VanA and the lead ancestral variants exist predominantly as monomers in solution under the in vitro conditions examined here. While this observation challenges the prevailing paradigm that Rieske-type *O*-demethylases require trimeric assembly to facilitate intersubunit electron transfer between the [2Fe–2S] cluster and the mononuclear iron center,^26^ it does not preclude a role for higher-order assembly in vivo. Indeed, trimeric architectures are a well-established feature of canonical Rieske oxygenases and may be conditionally stabilized by factors not captured in our assays, such as substrate binding and interaction with VanB. Alternatively, the predominance of a monomeric state in solution raises the possibility that VanAB systems employ an electron-transfer pathway that deviates from established models, highlighting the need for further studies to define both the mode of electron transfer within the oxygenase and its coupling to the VanB reductase.

To resolve the structural basis for the enhanced performance of *Anc*VanA3, we integrated deep-learning-based modeling with MD simulations. The MD trajectories revealed that the superior 3OMG conversion in *Anc*VanA3 likely arises from a wider substrate-binding pocket and the apparent loss of a gating mechanism found in the extant enzyme. In *Xac*VanA, MD simulations predicted a conformational change of the V300 residue that regulates active-site access, a mechanism that is evolutionarily convergent with the W285 gate in the DMO system.^27,52^ In *Anc*VanA3, this gating is disrupted, favoring a constitutively open conformation that facilitates substrate entry and product release (Figure 7). Furthermore, distal variations, such as the R294–D222 salt bridge in *Anc*VanA3, appear to influence these coordinated motions, reinforcing the idea that the catalytic outcomes of Rieske oxygenases are shaped by protein motifs far from the active site.^53^

Despite their superior performance in *E. coli*, these ancestral enzymes revealed a marked chassis dependence when evaluated in *P. putida* KT2440. *Anc*VanA3 failed to demethylate syringate or 3OMG in this host and appeared to induce a significant metabolic burden or toxicity, potentially due to its high expression levels and structural stability. These findings underscore that catalytic competence in one host does not guarantee functionality across others, suggesting that future efforts must employ strategies such as tuning expression levels,^54,55^ optimizing subunit stoichiometry,^56^ or using in vivo directed evolution^57^ to improve host compatibility.

In summary, this study successfully resurrected ancestral VanA variants with enhanced thermotolerance and reshaped catalytic profiles, providing a lead variant with superior activity toward the 3OMG bottleneck in lignin valorization. By demonstrating that these enzymes are predominantly monomeric, we provide a new conceptual framework for understanding electron transfer and assembly in Rieske *O*-demethylases. While host constraints remain a hurdle, the integration of ASR with mechanistic insights offers a robust strategy for advancing the engineering of VanAB systems for sustainable chemical production and lignin bioconversion.

## Methods

### Ancestral Sequence Reconstruction (ASR)

An initial profile Hidden Markov Model (HMM) was built from an alignment of Rieske *O*-demethylases, including the sequences of characterized vanillate *O*-demethylases, using the HMMER hmmbuild program (version 3.1b2).^58^ This profile was used in a broad search against the UniParc database, yielding ∼38,000 initial sequence hits. Multiple sequence alignments were generated with MAFFT v7.487,^59^ and preliminary phylogenetic trees were constructed to map known enzymatic activities to distinct clades. Subsequent sequence curation and phylogenetic inference was performed iteratively. During each iteration, refined profile HMMs were constructed from robust clades corresponding to known enzymatic activities and/or taxonomic designations. These profiles were used to update curated sequences by searches against UniParc.^60^ Resulting hits were clustered using MMseqs2 (version 15.6f452),^61^ followed by multiple sequence alignment and tree inference using IQ-TREE (version 2.1.2).^62^ This iterative approach increased phylogenetic coverage and improved clade resolution. Quality filtering and subsequent clustering at 99% identity yielded 1271 representative sequences. 141 sequences from a more distantly related, homologous clade were included as an outgroup for root placement. Phylogenetic inference of the final tree was conducted under the Jones–Taylor–Thornton (JTT) model of evolution, identified as the optimal model by IQ-TREE’s model finder. Four ancestral nodes were selected for experimental characterization. The most recent ancestral node N908, representing the ancestor of *Xanthomonas* (Proteobacteria) sequences including the extant target enzyme, was chosen and its correspondent enzyme designated as *Anc*VanA1. The ancestral node N143, predominantly constituted of Proteobacteria sequences, was chosen and its correspondent enzyme designated as *Anc*VanA2. Node N1020, corresponding to the ancestor of Actinobacteria sequences, was chosen and its correspondent enzyme designated as *Anc*VanA3. Finally, the node N141 in the phylogenetic tree was chosen as the oldest ancestor, representing a deep ancestor of all previous selected nodes, its correspondent enzyme was designated as *Anc*VanA4. Ancestral sequences were inferred with GRASP (Graphical Representation of Ancestral Sequence Predictions)^46^ using joint reconstruction by maximum likelihood, with site-specific substitution rates as generated by IQ-TREE. Marginal sequence reconstructions were performed to assess the robustness of ancestral inferences, identifying per-position probabilities for each of the selected ancestral sequences. Each ancestral node showed a high degree of agreement between the joint and marginal reconstructions. The amino acid sequence of the reconstructed ancestral variants is available in Table S2.

### Protein Expression and Purification

The genes for *Xac*VanA and *Xac*VanB were amplified by PCR using specific primers (Table S3) and cloned into pET28a­(+) and pET21b­(+), respectively, using the In-Fusion HD kit (Takara Bio, Kusatsu, Shiga, Japan). The ancestor oxygenases genes were codon-optimized for *E. coli*, synthesized and cloned into pET28a­(+) by GeneUniversal (Newark, DE, USA). *E. coli* BL21 (DE3) was cotransformed with pET28a::oxygenase and pISC (plasmid encoding the *E. coli* chaperones for iron–sulfur cluster assembly). The cells were cultured in LB medium with 50 μg/mL kanamycin and 34 μg/mL chloramphenicol at 37 °C, 200 rpm until OD600 nm ∼0.8. The protein expression was induced by adding 0.5 mM isopropyl-β-d-thiogalactopyranoside (IPTG) to the medium and incubating it at 18 °C for 16 h. The medium was supplemented with 0.5 mM ammonium ferric citrate at the same time of the induction. For each 500 mL volume expression, cells were harvested by centrifugation at 7500*g*, at 4 °C for 30 min, resuspended on 30 mL buffer A (25 mM HEPES, 400 mM NaCl, 10 mM imidazole, pH 7.5) containing cOmplete Mini protease inhibitor cocktail tablets (Roche, Basel, Switzerland) or 1 mM phenylmethanesulfonyl fluoride (PMSF), and 5 μg/mL DNase I (Merck, Darmstadt, Germany). Cells were disrupted by sonication (pulses of 15 s with intervals of 30 s during 15 min, 40% amplitude). The cell extract was centrifuged at 35250*g*, at 4 °C for 30 min. For the samples used in biophysical assays, the soluble fraction was incubated with 1 mL Ni–NTA agarose resin (QIAGEN, Hilden, Germany) under gentle agitation (4 °C, 2 h). The resin–protein mixture was then transferred into a gravity-flow column, washed with 30 mL buffer A to remove unbound proteins, washed additionally with 20 mL buffer B1 (25 mM HEPES, 400 mM NaCl, 50 mM imidazole, pH 7.5), and the target proteins were eluted with buffer B2 (25 mM HEPES, 400 mM NaCl, 250 mM imidazole, pH 7.5). Protein concentration was estimated based on the absorbance of protein samples at 280 nm using the extinction coefficient calculated from their amino acid sequences using ProtParam (Expasy server).^63^ For samples used in estimation of soluble protein yields, 500 mL of LB medium was used for expression under the same conditions described above. Purifications were performed as described above, except for the elution gradient. The fractions were eluted with buffer B1–B5 (imidazole gradient ranging from 50 to 500 mM) (Figure S1). Selected fractions were pooled and subjected to buffer exchange to remove imidazole using Econo-Pac 10DG columns (Bio-Rad Laboratories, Hercules, CA, USA). Next, the samples were concentrated ∼15 times using an Amicon filter (Merk). Protein concentrations were then determined using the dye-binding Bradford method (Bio-Rad Protein Assay Kit). The total amount of purified protein was calculated by multiplying the measured protein concentration by the final volume after buffer exchange. Yields were normalized to 1 L of culture medium.

### *E. coli* Whole-Cell Activity Assays

Whole-cell assays were inspired by the methodology described by García–Hidalgo et al. (2020).^64^ Briefly, *E. coli* BL21­(DE3) cells were cotransformed with pET28a::oxygenase (kanamycin resistance) and pET21b::reductase (ampicillin resistance). *Xac*VanB, an extant reductase from *X. citri*, was used as the reductase partner in all assays. Cells harboring the empty pET28a­(+) vector together with pET21b::*Xac*VanB were used as negative controls. Overnight cultures were grown in LB medium (10 mL, 37 °C, 250 rpm), harvested, washed, and inoculated to an initial OD_600_ of 0.4 into 125 mL Erlenmeyer flasks containing 10 mL of M9 medium (42.2 mM Na_2_HPO_4_, 22.0 mM KH_2_PO_4_, 8.6 mM NaCl, 18.7 mM NH_4_Cl, 1 mM MgSO_4_, 0.1 mM CaCl_2_, and 0.1 mM FeSO_4_), supplemented with 70 mM glucose, 50 μg/mL kanamycin, 100 μg/mL ampicillin, 0.5 mM IPTG and the desired substrate (5.0 mM for syringate and vanillate, and 4.3 mM for 3OMG). Aromatic substrate stock solutions were prepared in DMSO, resulting in a final DMSO concentration of 0.5% (v/v) in the culture medium. Cultures were incubated at 30 °C and 200 rpm for up to 24 h. Samples were collected at predetermined time points, centrifuged at 18000*g* for 10 min, and the supernatants were analyzed as described in the HPLC section. All assays were performed with three biological replicates.

### Differential Scanning Fluorimetry (DSF)

Protein thermal stability was assessed by differential scanning fluorimetry (DSF) in buffer 80 mM BICINE, pH 8.0 from JBScreen Solubility HTS (Jena Bioscience, Jena, Germany) containing 3–10 μM protein and SYPRO Orange protein gel stain (Thermo Fisher Scientific, Waltham, MA, USA) at a final concentration of 5× (from a 5000× stock). Fluorescence measurements were performed in a ViiA 7 Real-Time PCR System (Applied Biosystems, Waltham, MA, USA) using the ROX channel. Samples were subjected to a temperature ramp from 25 to 95 °C, with fluorescence acquisition at each 1 °C increment, monitoring the exposure of hydrophobic regions during thermal unfolding. Melting curves were fitted by Boltzmann sigmoidal regressions using Origin 2024 (OriginLab Corp., Northampton, MA, USA) and melting temperatures (*T*_m_) were determined from the inflection point of the unfolding transition. Each condition was analyzed in three replicates.

### Mass Photometry

Mass photometry measurements were performed using a TwoMP instrument (Refeyn Ltd., Oxford, UK), based on the interferometric scattering principle described by Young et al. (2018).^65^ All protein samples were analyzed in the same buffer conditions. Proteins were initially prepared in 25 mM HEPES, 400 mM NaCl, pH 7.5. Immediately prior to data acquisition, protein stocks at 100 nM were freshly prepared by dilution in 25 mM HEPES, 100 mM NaCl, pH 7.5, and subsequently introduced into the measurement coverslip to a final concentration of 10 nM. Data acquisition and initial analysis were carried out using the DiscoverMP software (Refeyn Ltd., Oxford, UK), which detects individual particle landing events and generates distributions based on interferometric contrast. For downstream analysis and figure preparation, raw contrast values were exported and used to generate histograms of normalized event counts. Peak positions, widths and relative populations were identified by the DiscoverMP software. Contrast-based histograms were replotted to improve graphical resolution using Origin 2024 (OriginLab Corp., Northampton, MA, USA), while preserving the original data and peak assignments. Theoretical molecular masses of the monomeric proteins were calculated from the protein sequences using ProtParam (Expasy server)^63^ and used to support the assignment of oligomeric species observed in the mass photometry measurements.

### SEC-MALS

Size-exclusion chromatography coupled to multiangle light scattering (SEC-MALS) was used to determine the oligomeric state of *Anc*VanA3. A total of 100 μL of the sample at 2 mg/mL was injected onto a Superdex 200 10/300 Increase size-exclusion column (Cytiva, Marlborough, MA, USA) using an Agilent 1260 Infinity II HPLC system (Agilent Technologies, Santa Clara, CA, USA). The system was equipped with a DAWN 8 multiangle light scattering detector and an Optilab refractive index detector (Wyatt Technology, Goleta, CA, USA). The mobile phase buffer consisted of 25 mM HEPES, 100 mM NaCl, pH 7.5, at a flow rate of 0.5 mL/min. Prior to sample analysis, the system was calibrated using bovine serum albumin (BSA) as a standard to verify detector performance and molecular weight accuracy. Data were analyzed using ASTRA 8.1.2 software (Wyatt Technology, Goleta, CA, USA).

### Bioconversion Assays in *P. putida* KT2440

Four *P. putida* KT2440 strains were constructed for bioconversion assays, each carrying a pSEVA2213 plasmid^66^ with an RK2 origin of replication containing distinct VanAB systems: (i) the native *vanA* and *vanB* genes from *P. putida* KT2440 (*Pp*VanAB), (ii) the gene encoding the ancestral oxygenase *Anc*VanA3 combined with the *Xac*VanB gene (*Anc*VanA3/*Xac*VanB), (iii) the *Xac*VanA and *Xac*VanB genes (*Xac*VanAB) and (iv) the corresponding empty vector used as a negative control. The VanAB systems were expressed under the control of the constitutive promoter PEM7. The *Anc*VanA3, *Xac*VanA and *Xac*VanB genes were codon-optimized for *P. putida* using the GenSmart Codon Optimization Tool (GenScript) and synthesized and cloned into pSEVA2213 plasmid by GenScript (Piscataway, NJ, USA). The native *vanA* and *vanB* genes were PCR-amplified from *P. putida* KT2440 chromosomal DNA using specific oligonucleotides (Table S3) and cloned into the pSEVA2213 plasmid using NEBuilder HiFi DNA Assembly (New England Biolabs, Ipswich, MA, USA). Overnight cultures were grown in LB medium (30 °C, 250 rpm), harvested, washed, and inoculated into 20 mL of culture medium dispensed in 125 mL Erlenmeyer flasks to an initial OD_600_ of 0.1. The culture medium consisted of a modified M9 minimal medium (42.2 mM Na_2_HPO_4_, 22.0 mM KH_2_PO_4_, 8.6 mM NaCl, 18.7 mM NH_4_Cl, 2 mM MgSO_4_, 0.1 mM CaCl_2_, 0.1 mM FeSO_4_, and 0.03% w/v casamino acids), supplemented with 40 mM glucose, 50 μg mL^–1^ kanamycin, and 5.5 mM syringate or 7.0 mM 3OMG. Aromatic substrate stock solutions were prepared in DMSO, resulting in a final DMSO concentration of 0.5% (v/v) in the culture medium. Cultures were incubated at 30 °C and 225 rpm. Samples were collected up to 48 h. At each time point, 2 mL culture aliquots were centrifuged (18000*g*, 10 min), and supernatants were analyzed by HPLC. Cell pellets were washed with 0.9% (w/v) NaCl saline solution and dried in a drying oven at 60 °C for 24 h prior to dry cell weight determination. All experiments were performed with three biological replicates.

### HPLC

High-performance liquid chromatography (HPLC) analyses were performed using an Agilent 1260 Infinity system equipped with a refractive index detector (RID) and a diode array detector (DAD). For *E. coli* whole-cell samples, the same methodology described by Martim et al. (2024)^25^ was used. Briefly, separations were performed using the Acclaim C18 column (150 mm × 4.6 mm) (Thermo Fisher Scientific, Waltham, MA, USA). The following conditions were applied: The mobile phases consisted of (A) 0.3% acetic acid in water and (B) methanol, with an analysis flow of 0.5 mL min^–1^ in the following gradient: *t* = 0 to 40 m in 15% (B) and 85% (A) to 30% (B) and 70% (A); *t* = 40 to 55 min: 30% (B) and 70% (A); *t* = 55 to 55.1 min: 30% (B) and 70% (A) to 15% (B) and 85% (A); *t* = 55.1 at 60 min 15% (B) and 85% (A). The total run time was 60 min. The column temperature was maintained at 30 °C and the DAD detector at 274 and 260 nm.

For *P. putida* KT2440 bioconversion samples, separations were performed using the Aminex HPX-87H column (300 mm × 7.8 mm) coupled to a guard column (30 mm × 4.6 mm) (Bio-Rad Laboratories, Hercules, CA, USA). The following conditions were applied: The mobile phases consisted of (A) 5 mM sulfuric acid in water and (B) 5 mM sulfuric acid containing 15% (v/v) acetonitrile, with an analysis flow of 0.7 mL min^–1^ in the following gradient: *t* = 0 to 0.1 min 100% (A) and 0% (B); *t* = 0.1 to 3 min: 100% (A) and 0% (B) to 37% (A) and 63% (B); *t* = 3 to 7 min: 37% (A) and 63% (B) to 25% (A) and 75% (B); *t* = 7 to 15 min: 25% (A) and 75% (B); *t* = 15.01 to 22 min: 0% (A) and 100% (B); *t* = 22.01 to 60 min 100% (A) and 0% (B). The total run time was 60 min. The column temperature was maintained at 65 °C, and the RID temperature was set at 55 °C.

### Comparative Sequence and Structural Analyses

Multiple sequence alignments to identify conserved Rieske motifs were performed using Expresso flavor of T-COFFEE web server^67,68^ and figures were generated in Jalview.^69^ Structural superimpositions were carried out using PyMOL v2.3 (The PyMOL Molecular Graphics System, Schrödinger LLC, New York, NY, USA). Protein–protein interface surface areas and free energy gains were calculated using PDBePISA.^48^ Three-dimensional protein structures were modeled using AlphaFold2 ^70^ or AlphaFold3,^49^ as indicated in the text. Intrinsic solubility scores were predicted using CamSol.^47^

### Molecular Dynamics (MD) Simulations

Protein structures (*Anc*VanA3 and *Xac*VanA) were modeled using the AlphaFold2.0 Colab notebook (ColabFold v1.5.5)^71^ and protonated at pH 7.5 using the H++ server.^72^ The atoms from the iron cluster ([2Fe–2S] and [Fe^3+^]) were added to the models using the AlphaFill server^73^ and optimized by quantum mechanics (QM) approach using the easyParm software.^74^ Geometry optimizations were carried out using the ORCA 6.0.1 quantum chemistry package at the B3LYP/6-31G­(d)^75^ level of theory using tight convergence thresholds for the Self-Consistent Field (SCF) approach. Constraints were applied on the protein side chains and atom caps. For the MD simulations, the Amber ff19SB force field^76^ was employed for protein, and each system was solvated using the TIP3P water molecules^77^ in a cubic box, ensuring a minimum distance of 15 Å between the protein and the box boundaries. Sodium ions (Na^+^) were added to neutralize both systems employing the Lennard–Jones (LJ) parameters optimized by Joung & Cheatham (2008).^78^ Initially, two consecutive energy minimizations using steepest descent algorithm were executed: one focused on the solvent and Na^+^ (with protein and ions restraint) followed by a minimization without any restraint to fully relax the system. Long-range interactions cutoff was defined as 12 Å using the particle mesh Ewald (PME) method. Four 1 ns heating steps were employed to increase the systems temperature to 303.15 K under the *NVT* ensemble followed by 10 ns NpT ensemble equilibration to adjust systems pressure to 1 bar. The production run using Amber24 package^79^ was executed in three independent simulations through 500- ns without restraint. Trajectory analyses were performed using Visual Molecular Dynamics (VMD) version 2.0,^80^ and pocket characterization throughout the trajectories was carried out with MDpocket,^81^ using the default parameters recommended for detecting small ligand-binding sites. Protein structure images were generated with PyMOL v2.3 (The PyMOL Molecular Graphics System, Schrödinger LLC, New York, NY, USA), and the MD analysis plots were produced in RStudio version 4.3.3 (Posit Software, Boston, MA, USA).

## Supplementary Material





## Data Availability

The codon-optimized nucleotide sequences encoding the characterized ancestral proteins have been deposited in the GenBank database under accession numbers: PZ060004 (*Anc*VanA1), PZ060005 (*Anc*VanA2), PZ060006 (*Anc*VanA3) and PZ060007 (*Anc*VanA4). *Xanthomonas citri* and other extant protein sequences were retrieved from the KEGG database [https://www.genome.jp] or from Uniprot [https://www.uniprot.org].

## Notes

Additional experimental results not included in the main text and supplementary tables with substrate conversion data and primer sequences (PDF)
Molecular dynamics visualization of key regions in *Xac*VanA (MP4)
