# Supplementary material for: Ancestral Protein Reconstruction Uncovers a Thermotolerant Rieske Oxygenase with Enhanced O‑Demethylation Activity toward 3‑O‑Methylgallate
Source: ACS Synth Biol. 2026 Jun 3;15(6):2660–74. doi: 10.1021/acssynbio.6c00325 (PMC13288931; doi:10.1021/acssynbio.6c00325)
Supplement: Supplementary file 1 [file sb6c00325_si_001.pdf]

Supplementary material

Ancestral Protein Reconstruction Uncovers a Thermotolerant Rieske Oxygenase with Enhanced *O*-demethylation Activity Toward 3-*O*-methylgallate

Augusto Rodrigues Lima[1,2], Gabriel Gonçalves Dias[1,2], Samuel J. Davis[3], Gabriela de Lima Menezes[1], Adriano Freitas Lima[1], Mariany da Silva Costa[1], Lúcia Daniela Wolf [1], Fernanda Miyuki Kashiwagi[1], Mariana Abrahão Bueno Morais[1], Mikael Bodén[3], Gerhard Schenk[3], and Priscila Oliveira de Giuseppe[1]

Affiliation:

[1]Brazilian Biorenewables National Laboratory (LNBR), Brazilian Center for Research in Energy and Materials (CNPEM), Campinas, SP 13083-100, Brazil.

[2]Graduate Program in Genetics and Molecular Biology (PPG-GBM), Institute of Biology, State University of Campinas (UNICAMP), Campinas, SP 13083-970, Brazil.

[3]School of Chemistry and Molecular Biosciences, The University of Queensland, St Lucia, QLD 4072, Australia.

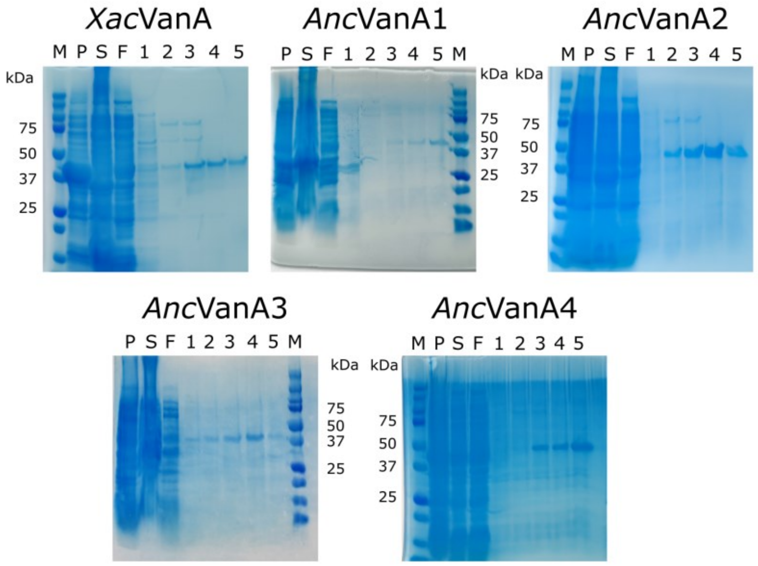

Figure S1. SDS-PAGE analysis of protein purification by imidazole gradient elution. All proteins were purified under the same conditions described in the Methodology section and eluted using the same buffer supplemented with increasing imidazole concentrations. Lane identification: M, molecular mass marker; P, insoluble pellet fraction; S, soluble fraction; F, flow-through. Lanes 1–5 correspond to fractions eluted with increasing imidazole concentrations: 1, 50 mM; 2, 100 mM; 3, 150 mM; 4, 250 mM; and 5, 500 mM imidazole. Fractions 3 to 5 were pooled (30 mL), buffer exchanged, centrifuged and concentrated to ~2 mL with Amicon filter (Merck). After this procedure, samples were collected, centrifuged, and protein concentrations were measured by a Bradford assay. Final estimated yields were as follows: X*ac*VanA, 5 mg/L culture; *Anc*VanA1, 11 mg/L culture; *Anc*VanA2, 20 mg/L culture; *Anc*VanA3, 34 mg/L culture; and *Anc*VanA4, 27 mg/L culture. Notably, the final estimate does not correlate with SDS–PAGE band intensities after IMAC, indicating protein precipitation during concentration.

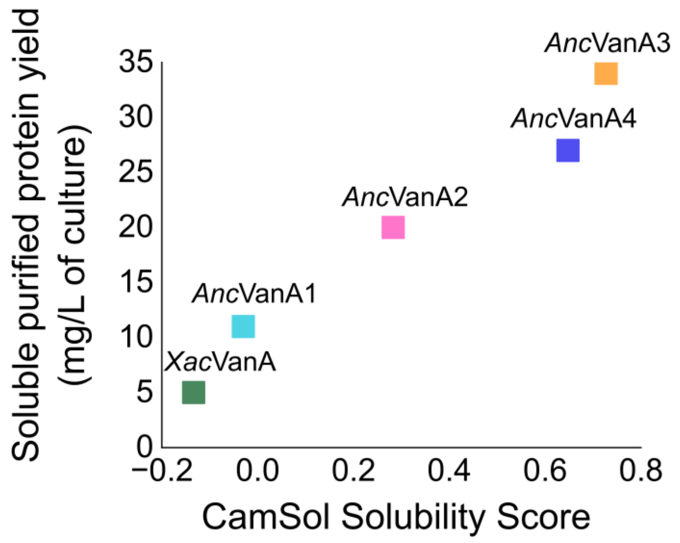

Figure S2. Correlation between intrinsic solubility scores and soluble protein yields. Protein sequences were used to predict the intrinsic solubility using CamSol[1]. The solubility scores were as follows: *Xac*VanA, -0.13; *Anc*VanA1, -0.03; *Anc*VanA2, 0.28; *Anc*VanA3, 0.73; and *Anc*VanA4, 0.65. Statistical analyses were performed using OriginPro 2024. Pearson's correlation analysis indicated a strong positive correlation ($r = 0.98$, $p = 0.002$). Spearman's rank correlation coefficient was $\rho = 1.0$ (exact monotonic correlation; p-value not computed due to the absence of rank variation).

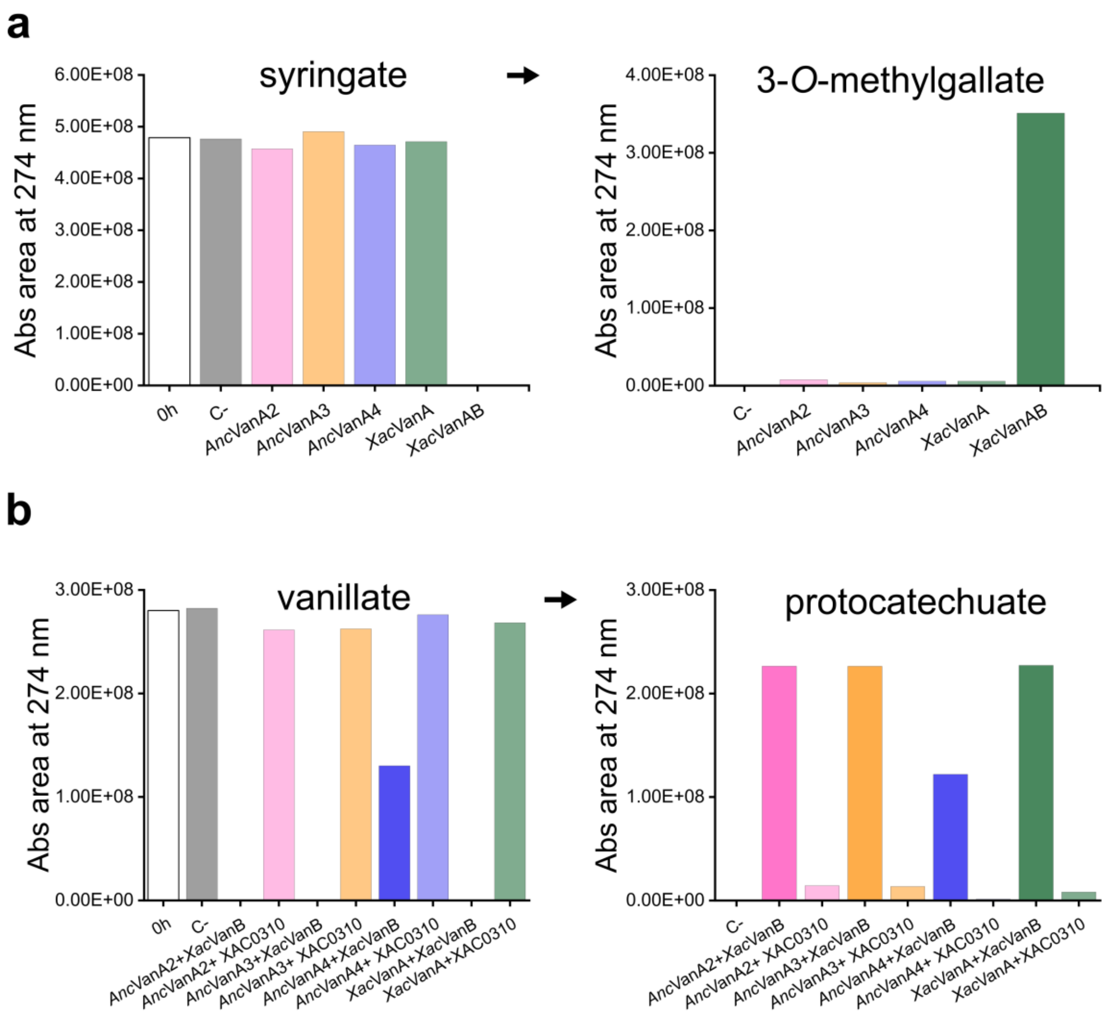

Figure S3. Testing the effect of reductase partners for the oxygenase activity. Whole-cell assays were performed using alternative *E. coli* constructs. (a) Cells were transformed with pET28a harboring the indicated oxygenase and an empty pET21b vector to evaluate whether native *E. coli* reductases could supply electrons to the oxygenase. The strain expressing *Xac*VanAB was used as a positive control. (b) VanA oxygenases were co-expressed either with *Xac*VanB or with the gene XAC0310, a paralogous VanB-like reductase from *Xanthomonas citri*. Note that all the oxygenases displayed activity only in the presence of *Xac*VanB. Data are presented as HPLC peak areas monitored at 274 nm, (n=1).

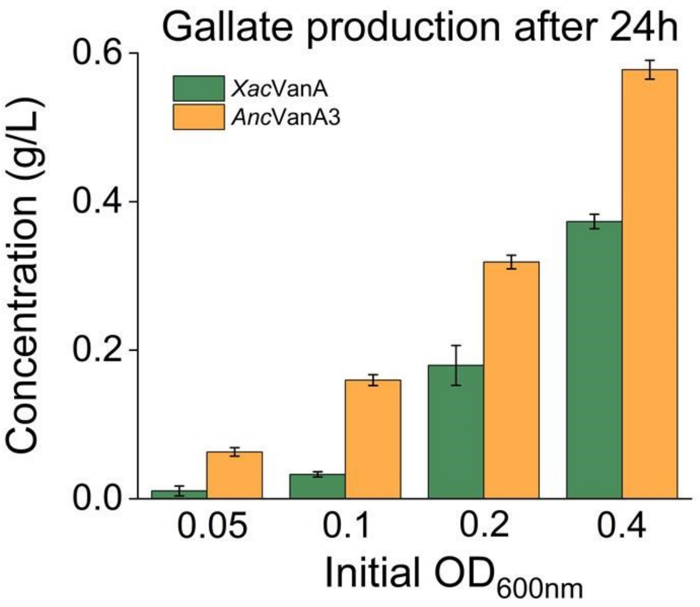

Figure S4. The ancestral enzyme *Anc*VanA3 outperformed *Xac*VanA regardless of the initial cell density. Under all tested conditions in *E. coli*, *Anc*VanA3 exhibited higher production of gallate (the product of 3OMG *O*-demethylation). Increasing the inoculum concentration (represented by OD600 nm), used as a proxy for enzyme concentration, leads to higher gallate production, as expected. Data are presented as mean ± standard deviation of triplicates.

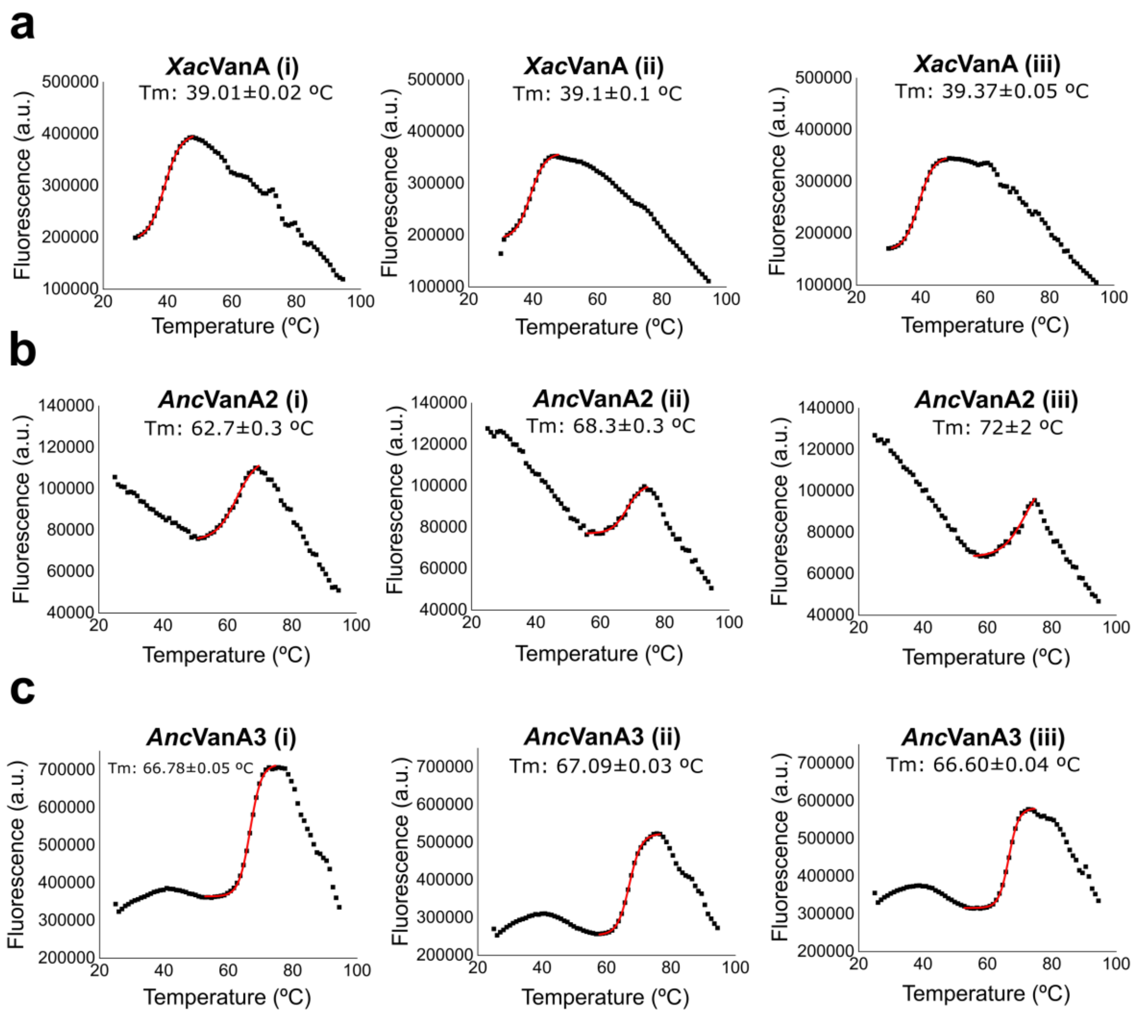

Figure S5. DSF profiles show the increased thermotolerance of ancestral variants. The melting temperature (T_m) was determined by sigmoidal regression (red line) applied to selected points of the melting curve derived from the measured Sypro Orange fluorescence (black dots). (a) *Xac*VanA three replicates, mean T_m= 39 ºC; (b) *Anc*VanA2 three replicates, mean T_m= 68 ºC; (c) *Anc*VanA3 three replicates, mean T_m= 67 ºC. All assays were performed using the same buffer (80 mM BICINE, pH 8.0). Regressions determined using Origin 2024 (OriginLab Corp.)

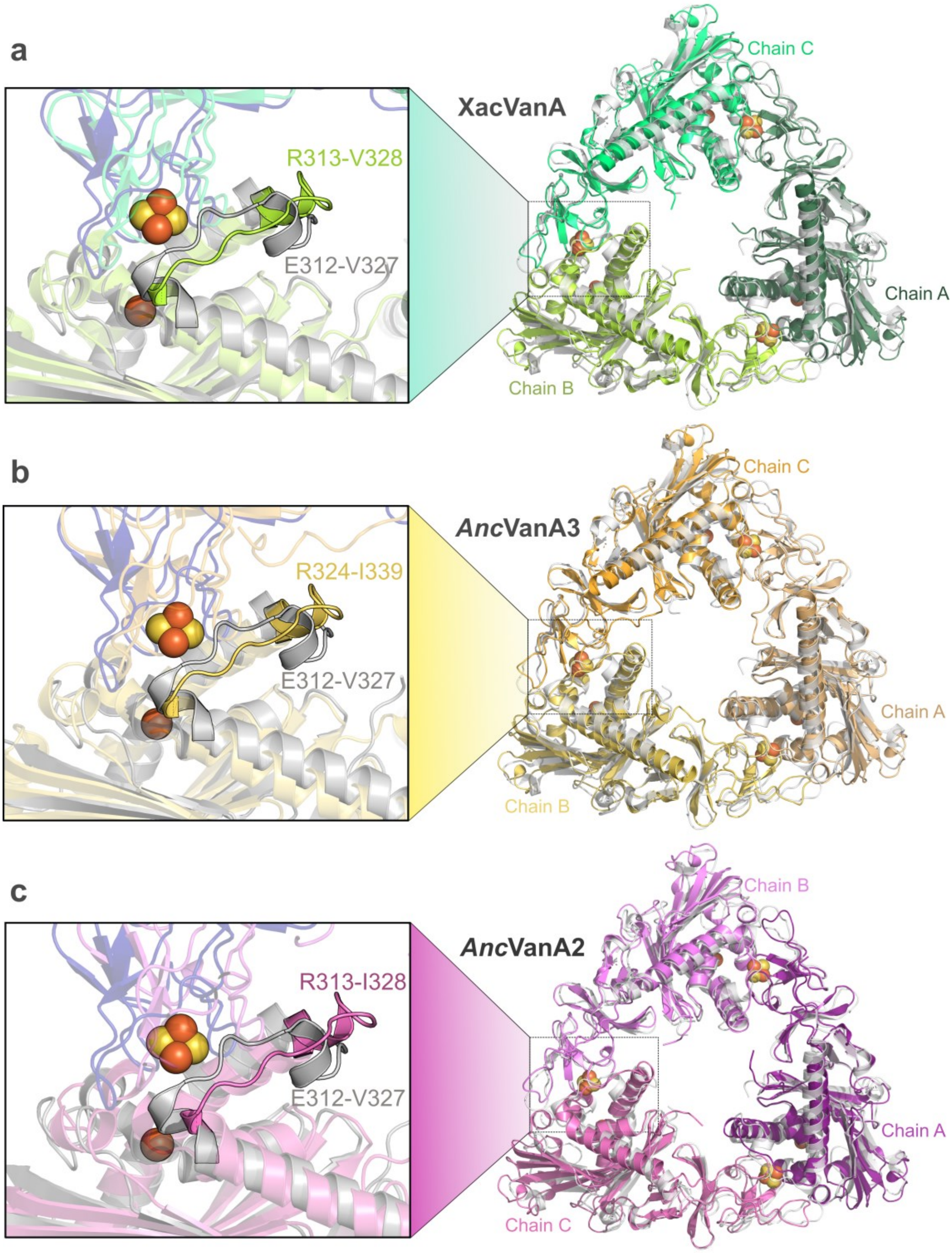

Figure S6. Analysis of protein-protein interfaces between adjacent protomers in trimeric models compared with the crystal structure of GdmA. All predicted trimeric models were superimposed onto the crystal structure of GdmA trimer (chains A, B, and C, PDB ID 7QWT, shown in gray). Selected interface regions are highlighted in the left panels, with close-up views of (a) *Xac*VanA (green color gradient), (b) *Anc*VanA3 (yellow color gradient), and (c) *Anc*VanA2 (pink color gradient). GdmA chain C is shown in blue (left panels). Spheres represent the crystallographic 2Fe–2S cluster and the catalytic iron from adjacent GdmA protomers.

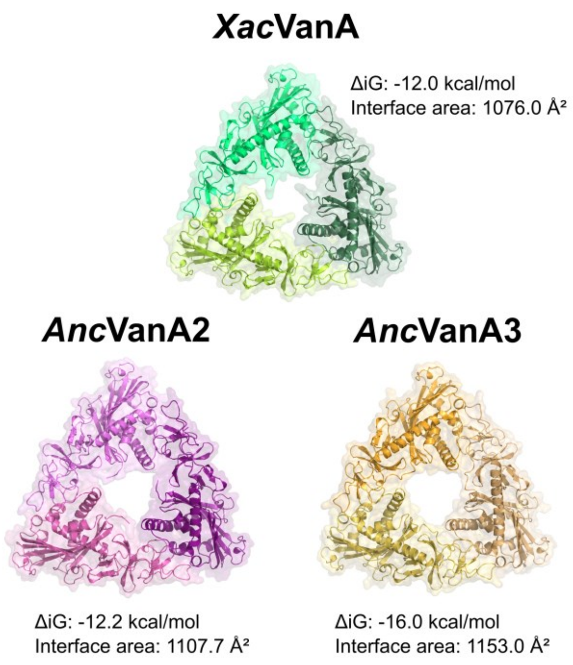

Figure S7. Predicted trimeric assemblies and interface analysis of *Xac*VanA, *Anc*VanA2 and *Anc*VanA3. Quaternary 3D structures were modeled with AlphaFold3, and figures of trimeric assemblies were generated using PyMOL. The interface surface area and the solvation free energy change associated with interface formation (ΔiG) were estimated using PDBePISA. Within each trimer, individual subunits are shown in cartoon representation using different shades of the same color, with transparent surface representations.

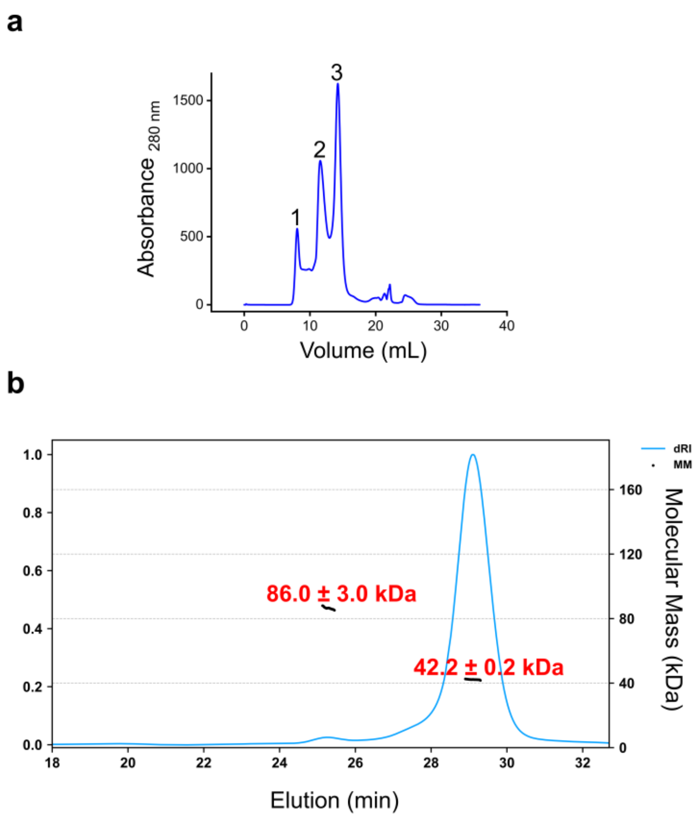

Figure S8. Oligomeric state analysis of *Anc*VanA3 by size-exclusion chromatography. (a) The SEC profile of *Anc*VanA3 was monitored at 280 nm, showing three major elution peaks. The central fraction of the elution peak 3 was collected and analyzed by SEC-MALS, as shown in panel b. (b) SEC-MALS analysis showed a predominant species with a molecular mass of 42.2 ± 0.2 kDa, consistent with a monomer, along with a minor population of 86 ± 3 kDa, indicative of dimeric species.

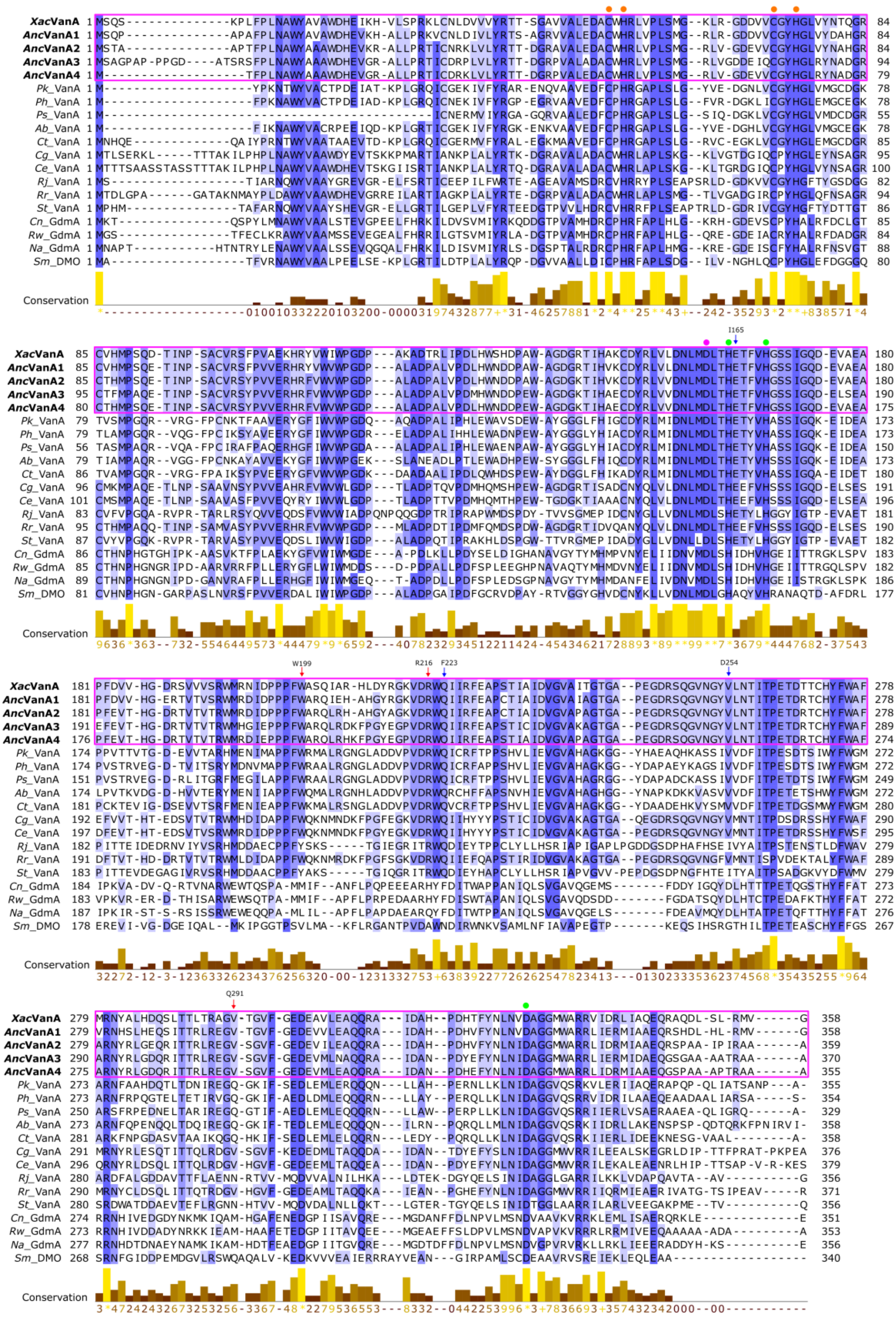

Figure S9. Multiple sequence alignment of characterized Rieske *O*-demethylases highlighting conserved motifs and possible substrate-specific residues in *Xac*VanA and ancestral variants. Orange circles: 2Fe-2S cluster binding motif. Green circles: 2Cys-1-carboxylate motif. Pink circle: gating keeper aspartate[2]. Arrows indicate possible residues

related to substrate specificity in VanA and GdmA homologs, according to molecular docking calculations conducted by Bleem et al. (2022)[2]. Red arrows: vanillate-specific residues, numbering according to the *Ph*_VanA sequence. Blue arrows: guaiacol-specific residues, numbering according to the *Rw*_GdmA sequence. Yellowish bars: bar height and color intensity (yellow) indicate the degree of amino acid conservation at each position. Amino acid residues are shown with a white-to-blue gradient indicating their degree of conservation (low to high) at each position. Species abbreviations correspond to the following organisms - *Xac*: *Xanthomonas citri*; *Anc*VanA1-A4: ancestral sequences reconstructed in this study; *Pp*: *Pseudomonas putida* KT2440; *Ph*: *Pseudomonas* sp. HR199; *Ps*: *Pseudomonas* sp. ATCC 19151; *Ab*: *Acinetobacter baylyi*; *Ct*: *Comamonas* sp.; *Cg*: *Corynebacterium glutamicum*; *Ce*: *Corynebacterium efficiens*; *Rj*: *Rhodococcus jostii*; *Rr*: *Rhodococcus ruber*; *St*: *Streptomyces* sp. NL15-2K; *Cn*: *Novosphingobium aromaticivorans*; *Rw*: *Rhizorhabdus wittichii*; *Na*: *Cupriavidus necator*; *Sm*: *Stenotrophomonas maltophilia.*

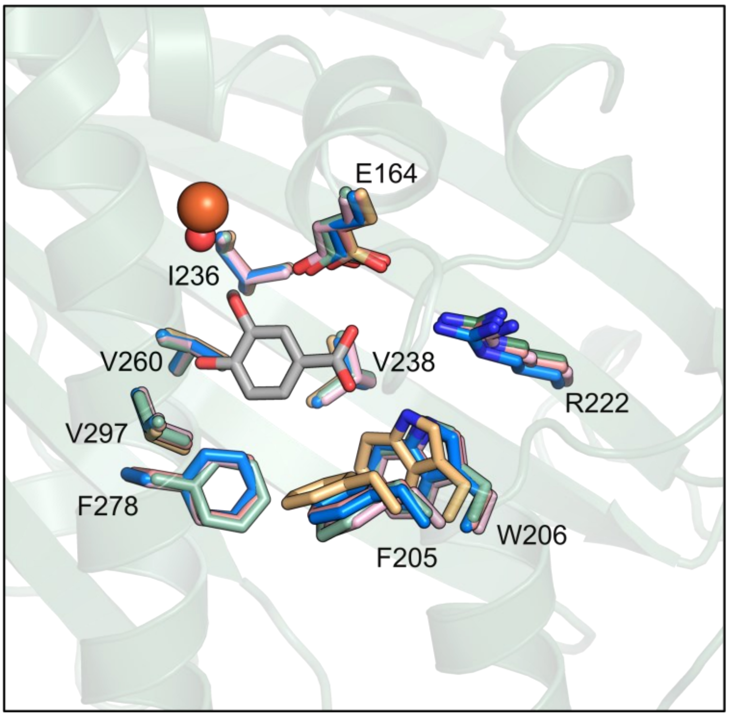

Figure S10. **Structural comparison of the predicted substrate binding pocket of VanA enzymes under investigation.** *Anc*VanA1 (cyan C atoms), *Anc*VanA2 (pink C atoms), *Anc*VanA3 (light orange C atoms), *Anc*VanA4 (blue-violet C atoms, occluded due to superposition), and *Xac*VanA (green C atoms). As a reference, a vanillate molecule was superimposed to this pocket, using the crystal structure of DMO in complex with dicamba as a reference (PDB ID 3GL2)[3]. Residues were labeled in reference to *Xac*VanA. Cartoon representation of *Xac*VanA 3D model superimposed to DMO (PDB ID 3GL2). The catalytic iron (orange sphere) and a crystallographic water (red sphere) are also shown.

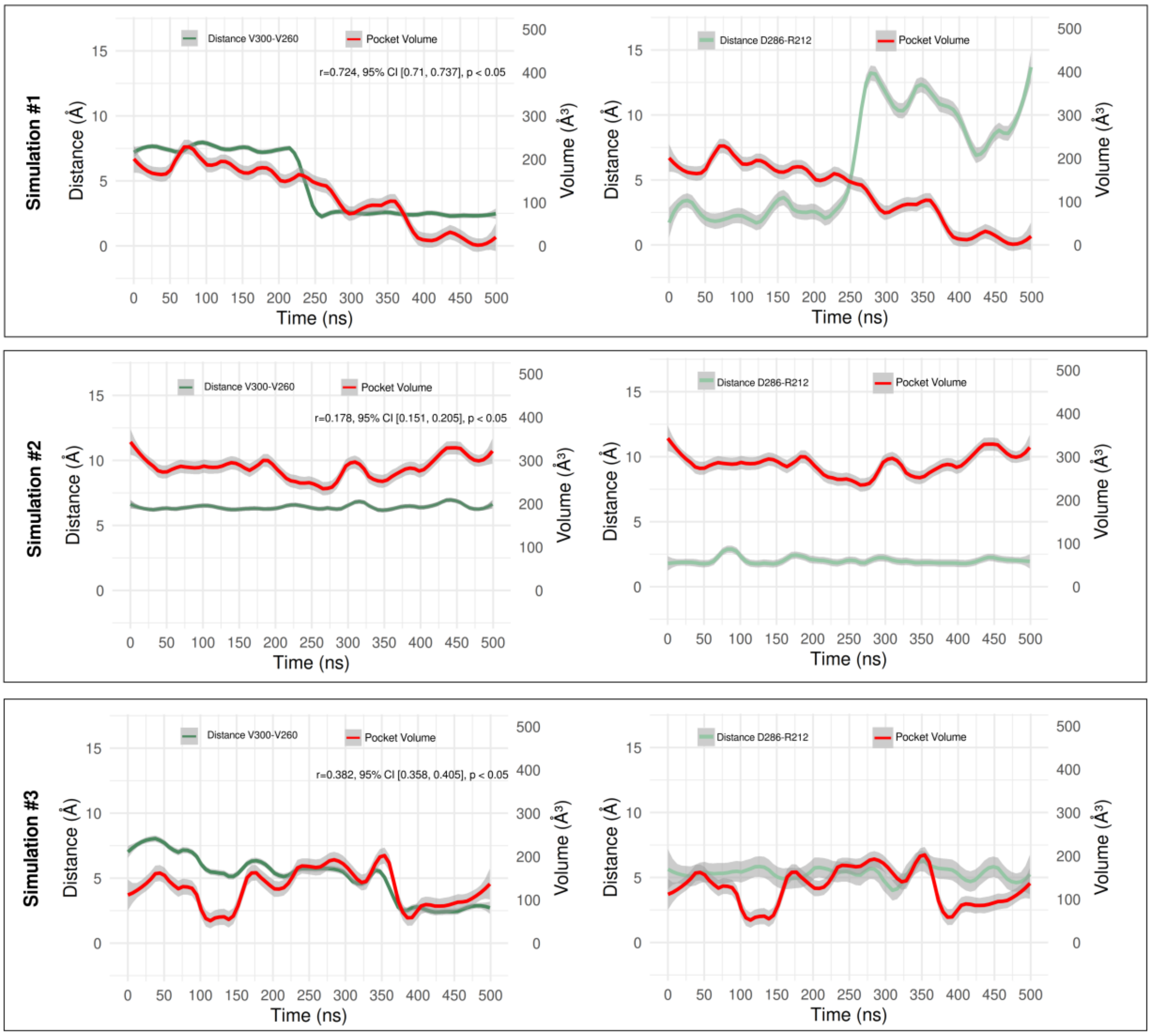

Figure S11. Amino acids pair distances and pocket volume during molecular dynamics simulations of *Xac*VanA. Left panels show the distance between V300-V260 and the pocket volume over MD simulation time in three independent simulations. V260 was used as the reference residue to determine the position of residue V300 relative to the pocket (inside or outside). Pearson correlation (r) analysis was performed to assess the statistical relationship between the residue-residue distance and pocket volume. Although the Pearson correlation of Replica 3 was 0.382 when considering entire trajectory, this value increases to 0.686 when the analysis is limited to the amino acid flip (between 335- to 400-ns), suggesting a strong correlation during this transition. Right panels show distance between D286 and R212 and pocket volume over MD simulation time in three independent simulations. These data suggest that the D286-R212 salt bridge interaction is possible associated with stability of the helix containing residue V300 and, consequently, with changes in pocket volume. All data were smoothed using the "geom_smooth" function from R "ggplot2" package[4]. The smoothing method applied was LOESS (locally estimated scatterplot smoothing) regression. Grey shaded areas represent the 95% confidence intervals.

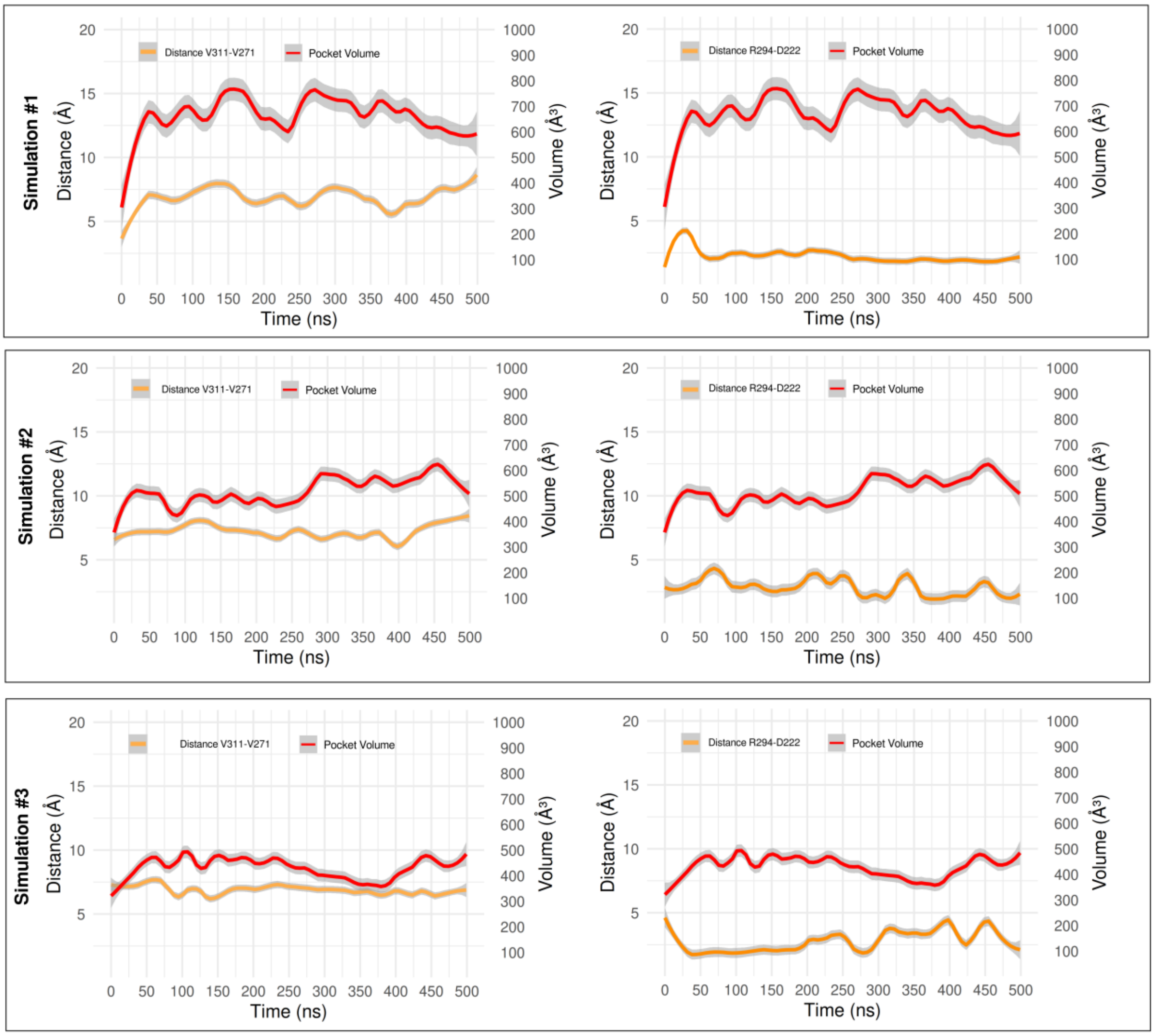

Figure S12. **Amino acids pair distances and pocket volume during 500-ns trajectory of *Anc*VanA3 protein.** Left panels show the distance between V311-V271 and the pocket volume over MD simulation time in three independent simulations. V271 was used as the reference residue to determine the position of residue V311 relative to the pocket (inside or outside). Right panels show the distance between D222-R294 and pocket volume over MD simulation time in three independent simulations. All data were smoothed using the "geom_smooth" function from R "ggplot2" package[4]. The smoothing method applied was LOESS (locally estimated scatterplot smoothing) regression. Grey shaded areas represent the 95% confidence intervals.

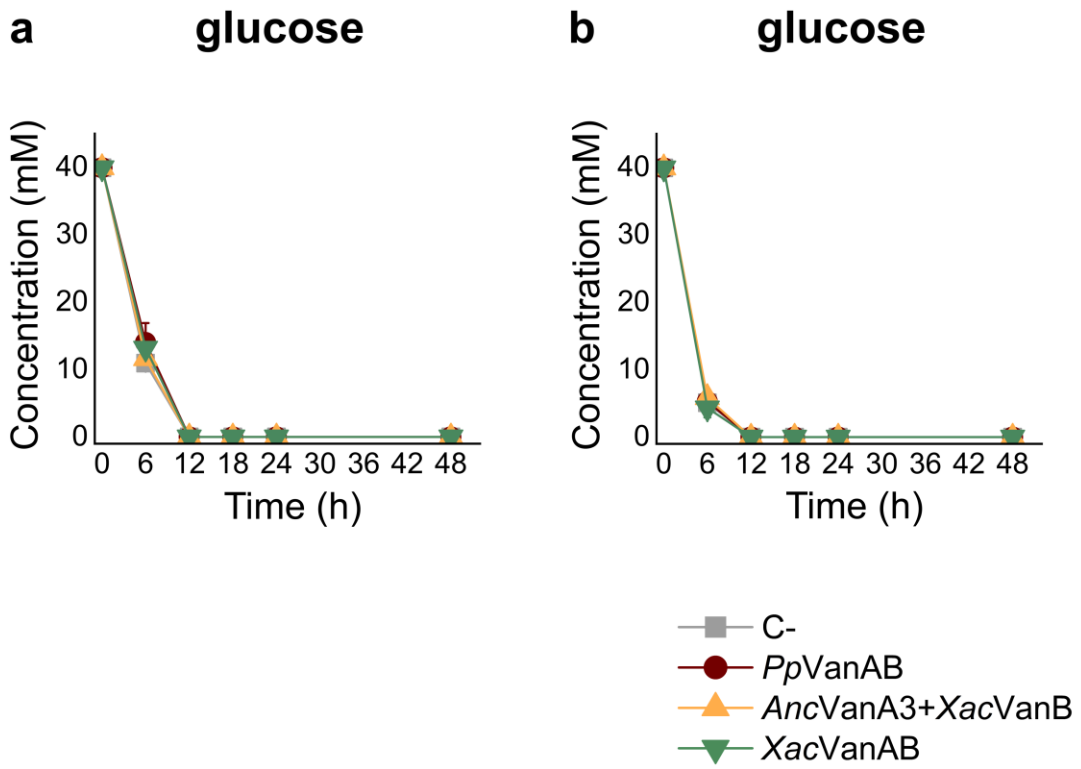

Figure S13. *Pseudomonas putida* KT2440 glucose consumption. The glucose was consumed similarly by all the different strains with (a) syringate or (b) 3-*O*-methylgallate as substrate. Data are presented as the mean ± SD of triplicates. C−: negative control corresponding to *P. putida* KT2440 transformed with the empty plasmid. *Pp*VanAB: *P. putida* KT2440 expressing its native *vanA* and *vanB* genes from a plasmid-borne construct. *Anc*VanA3 + *Xac*VanB: *P. putida* KT2440 expressing the ancestral oxygenase *Anc*VanA3 together with the extant reductase *Xac*VanB from a plasmid-borne construct. *Xac*VanAB: *P. putida* KT2440 expressing the extant oxygenase *Xac*VanA and its cognate reductase *Xac*VanB from a plasmid-borne construct.

Table S1 – HPLC-based quantification of substrate and products in *E. coli* whole-cell assays showing the estimated conversion rates from the initial substrate to the final demethylated product. The conversion rate (%) was calculated as the ratio of product concentration to initial substrate concentration, multiplied by 100. Uncertainties were propagated according to standard error propagation, assuming independent variables. No detected compounds are indicated by a dash.

**Oxygenase**	**Vanillate (mM) – 0h**	**Vanillate (mM) – 4h**	**Protocatechuate (mM) – 4h**	**Conversion rate (%)**
*Xac*VanA	4.86 ± 0.06	3.43 ± 0.11	1.29 ± 0.11	26 ± 2
*Anc*VanA1	4.86 ± 0.06	4.42 ± 0.05	0.22 ± 0.02	4.5 ± 0.5
*Anc*VanA2	4.86 ± 0.06	3.56 ± 0.07	1.12 ± 0.07	23 ± 1
*Anc*VanA3	4.86 ± 0.06	3.88 ± 0.08	0.75 ± 0.09	15 ± 2
*Anc*VanA4	4.86 ± 0.06	4.23 ± 0.06	0.41 ± 0.04	8 ± 1
C-	4.86 ± 0.06	4.66 ± 0.05	-	-
	**Vanillate (mM) – 0h**	**Vanillate (mM) – 8h**	**Protocatechuate (mM) – 8h**	**Conversion rate (%)**
*Xac*VanA	4.86 ± 0.06	0.79 ± 0.19	4.1 ± 0.2	85 ± 4
*Anc*VanA1	4.86 ± 0.06	3.79 ± 0.05	0.88 ± 0.04	18 ± 1
*Anc*VanA2	4.86 ± 0.06	0.41 ± 0.01	4.44 ± 0.01	91 ± 1
*Anc*VanA3	4.86 ± 0.06	1.41 ± 0.21	3.35 ± 0.24	69 ± 5
*Anc*VanA4	4.86 ± 0.06	3.0 ± 0.1	1.72 ± 0.13	35 ± 3
C-	4.86 ± 0.06	4.59 ± 0.04	-	-
	**Vanillate (mM) – 0h**	**Vanillate (mM) – 12h**	**Protocatechuate (mM) – 12h**	**Conversion rate (%)**
*Xac*VanA	4.86 ± 0.06	-	4.93 ± 0.01	101.4 ± 0.2
*Anc*VanA1	4.86 ± 0.06	3.16 ± 0.04	1.56 ± 0.03	32.1 ± 0.7
*Anc*VanA2	4.86 ± 0.06	-	4.89 ± 0.02	100.6 ± 0.4
*Anc*VanA3	4.86 ± 0.06	-	4.86 ± 0.03	100.0 ± 0.6
*Anc*VanA4	4.86 ± 0.06	1.7 ± 0.2	3.1 ± 0.2	64 ± 3
C-	4.86 ± 0.06	4.59 ± 0.01	-	-

	**Vanillate (mM) – 0h**	**Vanillate (mM) – 24h**	**Protocatechuate (mM) – 24h**	**Conversion rate (%)**
*Xac*VanA	4.86 ± 0.06	-	4.911 ± 0.004	101 ± 1
*Anc*VanA1	4.86 ± 0.06	0.55 ± 0.08	4.32 ± 0.04	89 ± 1
*Anc*VanA2	4.86 ± 0.06	-	4.87 ± 0.01	100 ± 1
*Anc*VanA3	4.86 ± 0.06	-	4.75 ± 0.05	98 ± 2
*Anc*VanA4	4.86 ± 0.06	-	4.81 ± 0.03	99 ± 1
C-	4.86 ± 0.06	4.59 ± 0.03	-	-
**Oxygenase**	**3OMG (mM) – 0h**	**3OMG (mM) – 4h**	**Gallate (mM) – 4h**	**Conversion rate (%)**
*Xac*VanA	4.36 ± 0.04	3.69 ± 0.02	0.341 ± 0.003	7.8 ± 0.1
*Anc*VanA1	4.36 ± 0.04	4.09 ± 0.01	0.025 ± 0.002	0.57 ± 0.05
*Anc*VanA2	4.36 ± 0.04	4.09 ± 0.01	0.097 ± 0.002	2.23 ± 0.05
*Anc*VanA3	4.36 ± 0.04	4.08 ± 0.03	0.069 ± 0.003	1.6 ± 0.1
*Anc*VanA4	4.36 ± 0.04	4.15 ± 0.04	0.009 ± 0.001	0.21 ± 0.02
C-	4.36 ± 0.04	4.14 ± 0.05	-	-
	**3OMG (mM) – 0h**	**3OMG (mM) – 8h**	**Gallate (mM) – 8h**	**Conversion rate (%)**
*Xac*VanA	4.36 ± 0.04	2.79 ± 0.05	1.31 ± 0.04	30 ± 1
*Anc*VanA1	4.36 ± 0.04	3.70 ± 0.03	0.40 ± 0.01	9.2 ± 0.2
*Anc*VanA2	4.36 ± 0.04	3.53 ± 0.01	0.64 ± 0.01	14.7 ± 0.3
*Anc*VanA3	4.36 ± 0.04	2.98 ± 0.03	1.16 ± 0.02	26.6 ± 0.5
*Anc*VanA4	4.36 ± 0.04	4.00 ± 0.05	0.17 ± 0.01	3.9 ± 0.2
C-	4.36 ± 0.04	4.07 ± 0.01	-	-
	**3OMG (mM) – 0h**	**3OMG (mM) – 12h**	**Gallate (mM) – 12h**	**Conversion rate (%)**
*Xac*VanA	4.36 ± 0.04	2.06 ± 0.07	1.99 ± 0.01	45.6 ± 0.5
*Anc*VanA1	4.36 ± 0.04	3.38 ± 0.03	0.72 ± 0.03	16.5 ± 0.7
*Anc*VanA2	4.36 ± 0.04	2.97 ± 0.05	1.09 ± 0.02	25.0 ± 0.5
*Anc*VanA3	4.36 ± 0.04	1.67 ± 0.02	2.30 ± 0.01	52.8 ± 0.5

*Anc*VanA4	4.36 ± 0.04	3.68 ± 0.01	0.45 ± 0.02	10.3 ± 0.5	
C-	4.36 ± 0.04	4.04 ± 0.03	-	-	
	**3OMG (mM) – 0h**	**3OMG (mM) – 24h**	**Gallate (mM) – 24h**	**Conversion rate (%)**	
*Xac*VanA	4.36 ± 0.04	0.63 ± 0.02	3.25 ± 0.02	74.5 ± 0.8	
*Anc*VanA1	4.36 ± 0.04	2.3 ± 0.1	1.80 ± 0.01	41.3 ± 0.4	
*Anc*VanA2	4.36 ± 0.04	1.80 ± 0.04	2.25 ± 0.02	52 ± 1	
*Anc*VanA3	4.36 ± 0.04	0.18 ± 0.01	3.66 ± 0.03	84 ± 1	
*Anc*VanA4	4.36 ± 0.04	2.44 ± 0.09	1.74 ± 0.09	40 ± 2	
C-	4.36 ± 0.04	3.96 ± 0.03	-	-	
**Oxygenase**	**Syringate (mM) – 0h**	**Syringate (mM) – 4h**	**3OMG (mM) – 4h**	**Gallate (mM) – 4h**	**Conversion rate (%)**
*Xac*VanA	4.76 ± 0.07	1.71 ± 0.02	2.62 ± 0.02	0.0143 ±0.0003	0.30 ± 0.01
*Anc*VanA1	4.76 ± 0.07	3.8 ± 0.1	0.692 ± 0.003	0.0068 ±0.0001	0.14 ± 0.01
*Anc*VanA2	4.76 ± 0.07	2.6 ± 0.1	1.9 ± 0.1	0.017 ±0.005	0.4 ± 0.1
*Anc*VanA3	4.76 ± 0.07	3.58 ± 0.04	0.93 ± 0.02	0.005 ±0.0003	-
*Anc*VanA4	4.76 ± 0.07	4.28 ± 0.01	0.21 ± 0.01	-	-
C-	4.76 ± 0.07	4.5 ± 0.1	-	-	-
	**Syringate (mM) – 0h**	**Syringate (mM) – 8h**	**3OMG (mM) – 8h**	**Gallate (mM) – 8h**	**Conversion rate (%)**
*Xac*VanA	4.76 ± 0.07	-	3.71 ± 0.09	0.79 ±0.01	16.6 ± 0.3
*Anc*VanA1	4.76 ± 0.07	1.7 ± 0.3	2.6 ± 0.2	0.06 ±0.01	1.3 ± 0.2
*Anc*VanA2	4.76 ± 0.07	0.01 ± 0.01	4.20 ± 0.04	0.27 ±0.04	5.7 ± 0.8
*Anc*VanA3	4.76 ± 0.07	0.023 ± 0.004	4.08 ± 0.05	0.30 ±0.01	6.3 ± 0.3
*Anc*VanA4	4.76 ± 0.07	3.71 ± 0.07	0.78 ± 0.12	-	-
C-	4.76 ± 0.07	4.44 ± 0.02	-	-	-
	**Syringate (mM) – 0h**	**Syringate (mM) – 12h**	**3OMG (mM) – 12h**	**Gallate (mM) – 12h**	**Conversion rate (%)**
*Xac*VanA	4.76 ± 0.07	-	2.87 ± 0.05	1.58 ±0.04	33 ± 1

*Anc*VanA1	4.76 ± 0.07	0.3 ± 0.3	3.9 ± 0.2	0.3 ±0.1	6 ± 2
*Anc*VanA2	4.76 ± 0.07	-	3.8 ± 0.1	0.8 ±0.1	17 ± 2
*Anc*VanA3	4.76 ± 0.07	-	2.72 ± 0.07	1.80 ±0.04	38 ± 1
*Anc*VanA4	4.76 ± 0.07	3.0 ± 0.2	1.5 ± 0.1	0.011 ±0.002	0.23 ± 0.04
C-	4.76 ± 0.07	4.49 ± 0.04	--	-	-
	**Syringate (mM) – 0h**	**Syringate (mM) – 24h**	**3OMG (mM) – 24h**	**Gallate (mM) – 24h**	**Conversion rate (%)**
*Xac*VanA	4.76 ± 0.07	-	0.94 ± 0.05	3.6 ±0.1	76 ± 3
*Anc*VanA1	4.76 ± 0.07	-	2.30 ± 0.02	2.26 ±0.02	47.5 ± 0.8
*Anc*VanA2	4.76 ± 0.07	-	2.5 ± 0.3	2.1 ±0.3	45 ± 6
*Anc*VanA3	4.76 ± 0.07	-	0.23 ± 0.03	4.39 ±0.09	92 ± 2
*Anc*VanA4	4.76 ± 0.07	-	4.06 ± 0.01	0.48 ±0.07	10 ± 1
C-	4.76 ± 0.07	4.48 ± 0.05	-	-	-

Table S2 – Ancestral Rieske *O*-demethylases sequences

name	aminoacids sequence
*Anc*VanA1	MSQPAPAFPLNAWYAVAWDHEVKHALLPRKVCNLDIVLYRTSAGRVVALEDACWHRLVPLSMGRLRGDDVVCGYHG<br>LVYDAHGRCVHMPSQETINPSACVRSFPVVERHRYVWVWPGDPALADPALVPDLHWNDDPAWAGDGRTIHVKCDYR<br>LVLDNLMDLTHETFVHGSSIGQDEVAEAPFDVVHGERTVTVSRWMRDIDPPPFWARQIEHAHGYRGKVDRWQIIRF<br>EAPCTIAIDVGVAIAGTGAPEGDRSQGVNGYVLNTITPETDRTCHYFWAFVRNHSLHEQSITTRLREGVTGVFGED<br>EVVLEAQQRAIDAHPDHVFYNLNVDAGGMWARRLIERMIAAEQRSHDLHLRMVG
*Anc*VanA2	MSTAAPTFPLNAWYAAAWDHEVKRALLPRTICNRKLVLYRTTDGRAVALEDACWHRLVPLSMGRLVGDEVVCGYHG<br>LVYNAHGRCTHMPSQETINPSACVRSYPVVERHRFVWVWPGDPALADPALVPDLHWNDDPAWAGDGKTIHVKCDYR<br>LVVDNLMDLTHETFVHGSSIGQDEVAEAPFEVTHGDRTVTVTRWMRDIDPPPFWARQLRHAHGYAGKVDRWQIIRF<br>EAPCTIAIDVGVAPAGTGAPEGDRSQGVNGYVLNTITPETDRTCHYFWAFARNYRLGEQRITTRLREGVSGVFGED<br>EVILEAQQRAIDAHPDHVFYNLNIDAGGMWARRLIDRMIAAEQRSPAAIPIRAAA
*Anc*VanA3	MSAGPAPPPGDATSRSFPLNAWYAAAWDHEVGRALLPRTICDRPLVLYRTTDGRPVALADACWHRLVPLSMGRLVG<br>DDEIQCGYHGLRYNADGRCTFMPAQETINPSACVRSYPVVERHRFVWVWPGDPALADPALVPDMHWNDDPEWAGDG<br>KTIHAECDYRLVVDNLMDLTHETFVHGSSIGQDELSEAEFEVTHGDRTVTVTRWMHGIEPPPFWARQLRDKFPGYE<br>GPVDRWQIIRFEAPSTICIDVGVAKAGTGAPEGDRSQGVNGYVLNTITPETDRTCHYFWAFARNYRLGDQRITTRL<br>REGVSGVFGEDEVMLNAQQRAIDANPDYEFYNLNIDAGGMWARRLIERMIDAEQGSGAAAATRAAA
*Anc*VanA4	MTFPLNAWYAAAWDHEVGRALLPRTICDRKLVLYRTTDGRPVALEDACWHRLVPLSMGRLVGDEVVCGYHGLRYNA<br>DGRCTHMPSQETINPSACVRSYPVVERHRFVWVWPGDPALADPALVPDLHWNDDPEWAGDGKTIHAECDYRLVVDN<br>LMDLTHETFVHGSSIGQDEVAEAPFEVTHGDRTVTVTRWMRDIEPPPFWARQLRHKFPGYEGPVDRWQIIRFEAPS<br>TIAIDVGVAPAGTGAPEGDRSQGVNGYVLNTITPETDRTCHYFWAFARNYRLGDQRITTRLREGVSGVFGEDEVML<br>EAQQRAIDANPDHEFYNLNIDAGGMWARRLIDRMIAAEQGSPAAAPTRAAA

Table S3 – Oligonucleotide primers. F = forward. R = reverse.

Primer	Gene	Sequence 5'-3'
363_F	vanA (*X. citri*)	CGCGCGGCAGCCATATGTCGCAGTCCAAGCCG
363_R	vanA (*X. citri*)	GACGGAGCTCGAATTCTTAGCCCACCATGCGCAAC
362_F	vanB (*X. citri*)	AAGGAGATATACATATGCGTAAAGACACCCAGTGGC
362_R	vanB (*X. citri*)	GGTGGTGGTGCTCGAGGCCCAGCTCGCTGCGATAGC
Pp_3736_F	vanA (*P. putida* KT2440)	CGCGAATTCGAGCTCGGTACTGACCTAAGGAGGTAAATAATGTACCCCAAAAACACCTGGTACGTC
Pp_3736_R	vanA (*P. putida* KT2440)	TCAGGCAGGGTTGGCGCTG
Pp_3737_F	vanB (*P. putida* KT2440)	CCAGCGCCAACCCTGCCTGATGACCTAAGGAGGTAAATAATGATCGATGCCGTAGTGG
Pp_3737_R	vanB (*P. putida* KT2440)	CAACAGGAGTCCAAGACTAGTCAGATGTCCAGCACCAGC

Supplementary references

(1) Sormanni, P., Aprile, F. A., and Vendruscolo, M. (2015) The CamSol Method of Rational Design of Protein Mutants with Enhanced Solubility. J. Mol. Biol. 427, 478–490.

(2) Bleem, A., Kuatsjah, E., Presley, G. N., Hinchen, D. J., Zahn, M., Garcia, D. C., Michener, W. E., König, G., Tornesakis, K., Allemann, M. N., Giannone, R. J., McGeehan, J. E., Beckham, G. T., and Michener, J. K. (2022) Discovery, characterization, and metabolic engineering of Rieske non-heme iron monooxygenases for guaiacol O-demethylation. *Chem Catalysis 2*, 1989–2011.

(3) Dumitru, R., Jiang, W. Z., Weeks, D. P., and Wilson, M. A. (2009) Crystal Structure of Dicamba Monooxygenase: A Rieske Nonheme Oxygenase that Catalyzes Oxidative Demethylation. *J. Mol. Biol. 392*, 498–510.

(4) Wickham, H. (2016) ggplot2: Elegant Graphics for Data Analysis 2nd ed. Springer International Publishing AG, Cham, Switzerland.
